# European Headache Federation (EHF) consensus on the definition of effective treatment of a migraine attack and of triptan failure

**DOI:** 10.1186/s10194-022-01502-z

**Published:** 2022-10-12

**Authors:** Simona Sacco, Christian Lampl, Faisal Mohammad Amin, Mark Braschinsky, Christina Deligianni, Derya Uludüz, Jan Versijpt, Anne Ducros, Raquel Gil-Gouveia, Zaza Katsarava, Paolo Martelletti, Raffaele Ornello, Bianca Raffaelli, Deirdre M. Boucherie, Patricia Pozo-Rosich, Margarita Sanchez-del-Rio, Alexandra Sinclair, Antoinette Maassen van den Brink, Uwe Reuter

**Affiliations:** 1 Department of Biotechnological and Applied Clinical Sciences, University of L’Aquila, Via Vetoio 1, L’Aquila, Italy; 2 Department of Neurology, Headache Medical Center at the Konventhospital BHB Linz, Linz, Austria; 3 Danish Headache Center, Department of Neurology, Rigshospitalet Glostrup, University of Copenhagen, Copenhagen, Denmark; 4 Department of Neurorehabilitation/Traumatic Brain Injury, Rigshospitalet, University of Copenhagen, Copenhagen, Denmark; 5 Department of Neurology, Institute of Clinical Medicine, University of Tartu; Headache Clinic, Department of Neurology, Tartu University Hospital, Tartu, Estonia; 6 Department of Neurology Istanbul Cerrahpasa Medical Faculty, Istanbul, Turkey; 7 Department of Neurology, Vrije Universiteit Brussel (VUB), Universitair, Ziekenhuis Brussel, Brussels, Belgium; 8 Neurology Department, CHU de Montpellier Charles Coulomb Laboratory, Montpellier University, Montpellier, France; 9 Neurology Department, Hospital da Luz Headache Center, Hospital da Luz, Lisbon, Portugal; 10 Center for Interdisciplinary Research in Health, Universidade Católica Portuguesa, Lisbon, Portugal; 11 Christian Hospital, Unna, Germany; 12 University of Duisburg-Essen, Essen, Germany; 13 Department of Clinical and Molecular Medicine, Sapienza University, Rome, Italy; 14 Department of Neurology, Charité Universitätsmedizin Berlin, Berlin, Germany; 15 Division of Vascular Medicine and Pharmacology, Department of Internal Medicine, Erasmus University Medical Center, Rotterdam, The Netherlands; 16 Headache Unit, Neurology Department, Vall d’Hebron University Hospital, Barcelona, Spain; 17 Department of Medicine, Headache and Neurological Pain Research Group, Vall d’Hebron Research Institute, Universitat Autònoma de Barcelona, Barcelona, Spain; 18 Department of Neurology, Clinica Universidad de Navarra, Madrid, Spain; 19 Institute of Metabolism and Sytems Research, University of Birmingham, Birmingham, UK; 20 Department of Neurology, University Hospitals Birmingham NHS Foundation Trust, Queen Elizabeth Hospital, Birmingham, UK; 21 Department of Internal Medicine, Erasmus MC Medical Center, Rotterdam, The Netherlands; 22 Universitätsmedizin Greifswald, Greifswald, Germany

**Keywords:** Migraine, Headache, Attack, Triptan, Gepant, Ditan, NSAIDs

## Abstract

**Background:**

Triptans are migraine-specific acute treatments. A well-accepted definition of triptan failure is needed in clinical practice and for research. The primary aim of the present Consensus was to provide a definition of triptan failure. To develop this definition, we deemed necessary to develop as first a consensus definition of effective treatment of an acute migraine attack and of triptan-responder.

**Main body:**

The Consensus process included a preliminary literature review, a Delphi round and a subsequent open discussion. According to the Consensus Panel, *effective treatment of a migraine attack* is to be defined on patient well-being featured by a) improvement of headache, b) relief of non-pain symptoms and c) absence of adverse events. An attack is considered effectively treated if patient’s well-being, as defined above, is restored within 2 hours and for at least 24 hours. An individual with migraine is considered as *triptan-responder* when the given triptan leads to effective acute attack treatment in at least three out of four migraine attacks. On the other hand, an individual with migraine is considered triptan non-responder in the presence of failure of a single triptan (not matching the definition of triptan-responder). The Consensus Panel defined an individual with migraine as *triptan-resistant* in the presence of failure of at least 2 triptans; *triptan refractory*, in the presence of failure to at least 3 triptans, including subcutaneous formulation; *triptan ineligibile* in the presence of an acknowledged contraindication to triptan use, as specified in the summary of product characteristics.

**Conclusions:**

The novel definitions can be useful in clinical practice for the assessment of acute attack treatments patients with migraine. They may be helpful in identifying people not responding to triptans and in need for novel acute migraine treatments. The definitions will also be of help in standardizing research on migraine acute care.

**Supplementary Information:**

The online version contains supplementary material available at 10.1186/s10194-022-01502-z.

## Background

Triptans are serotonin agonists acting on the 5-HT_1B/1D(/1F)_ receptors, which were specifically designed for the acute treatment of migraine. Their vasoconstrictive properties are mediated by the 5-HT_1B_ receptor present in arterial smooth muscles [1]. Activation of 5-HT_1D_ receptor on trigeminal fibers inhibits the release of peripheral vasoactive neuropeptides such as substance P and calcitonin gene-related peptide (CGRP). Some triptans can, with varying affinity, activate the 5-HT_1F_ receptor, which also inhibits CGRP release and possibly nociceptive modulation and dural neurogenic inflammation [2, 3]. Apart from their 5-HT_1F_ receptor affinity, all triptans have similar pharmacodynamics, although second generation triptans (almotriptan, eletriptan, frovatriptan, naratriptan, rizatriptan, and zolmitriptan) were designed to have an improved pharmacokinetic profile compared with sumatriptan, including a higher oral bioavailability combined with a comparable or increased plasma half-life or faster rapid onset of action [4, 5]. In general, triptans are presumed too hydrophilic to cross the blood-brain barrier and thus to have a central effect.

The clinical introduction of sumatriptan was a breakthrough for acute migraine treatment. To date, seven triptans in different formulations have demonstrated their superiority to placebo in clinical trials and are currently available for moderate to severe migraine attacks [6]. Triptans are widely prescribed not only in headache centers, and general neurology but also in primary care [7].

In randomized clinical trials (RCTs), several endpoints have been used to assess triptan efficacy and, consequently, response rates. Most trials focused on one single migraine attack and considered substantial pain relief or pain freedom after 1 to 4 h as an adequate response. Despite the general good efficacy, almost one-third of individuals with migraine did not meet such endpoints across trials [8]. However, the lack of response to one triptan during one single attack is not sufficient to determine a general poor response to the whole medication class. In fact, individuals with migraine might respond to the same triptan during a different attack, or to a higher dose and a different formulation of the same triptan or another triptan type. Other factors, such as the timing of dosing relative to the attack onset, accompanying nausea/vomiting, or medication overuse can also contribute to the differences in response [9].

A general definition of triptan failure does not exist. From a clinical point of view, defining criteria for non-response could have several benefits:

1. Create a standardized algorithm for migraine acute treatment in case of failure to one triptan, i.e. recommendations of which other triptans could lead to a positive effect;
2. Identify a population of non-responders to triptans and provide treatment guidelines in these cases;
3. Standardize definitions for clinical trials to investigate, which factors are associated with triptan failure.

Evidence on the precise link between pharmacodynamics, pharmacokinetics, and clinical response to triptans is limited and hindered by inconsistent findings. When comparing responders with non-responders, one study observed that absorption of oral sumatriptan was lower and slower in unsatisfactory responders [10], while other studies found no differences in pharmacokinetics nor genetic diversity of the 5-HT_1B_ or 5-HT_1F_ receptor [11, 12] after oral or subcutaneous sumatriptan. Yet, some findings do suggest that pharmacokinetics plays a role: the lack of response to one triptan does not predict responsiveness to another triptan [13], and the highest response rates are observed after subcutaneous sumatriptan. Of interest is a systematic review and meta-analysis which found that women have a higher drug exposure compared with men, without their response being higher [14].

Factors of importance for headache response to triptans may include time of administration and rapid onset of action, such as observed with intranasal or subcutaneous administration [10, 15]. Longer half-life and higher 5-HT_1B_ receptor potency also influence effectiveness as they are associated with lower headache recurrence [16]. Predictors of response to triptans also include the normalization of elevated CGRP levels in saliva or the extra-jugular vein after drug administration [17, 18], but due to high inter-individual variability, such predictors are not yet useful in the clinical context [19].

The primary aim of the present Consensus was to provide a definition of triptan failure to be used in clinical practice and research. We acknowledge that definition of triptan failure cannot be developed in the absence of a definition of effective treatment of an acute migraine attack and of a definition of triptan-responder and for this reason we agreed upon those definitions before establishing a consensus definition of triptan failure.

## Methods

### Preliminary systematic review

To inform the decisions of the consensus group, we performed a systematic review based on the Preferred Reporting Items for Systematic reviews and Meta-Analyses (PRISMA) guidelines [20]. We launched a database search on PubMed, Scopus, and Web of Science on October 5th, 2021, with the following search string: “(triptan* OR almotriptan OR eletriptan OR frovatriptan OR naratriptan OR rizatriptan OR sumatriptan OR zolmitriptan) AND migrain* AND response*”. We applied filters for English language and human studies. We selected RCTs and observational studies investigating the efficacy and effectiveness of triptans (at any dose and any formulation) in individuals with migraine. We excluded studies not performed in individuals with migraine (such as Phase I trials on healthy subjects), non-original studies (such as editorials, letters, commentaries, and reviews), and studies without data of interest for this work (such as those with economic evaluations or only safety data).

Two researchers (RO and BR) independently assessed titles and abstracts for eligibility; disagreements were resolved by consensus. After that, full texts were screened for eligibility in qualitative synthesis by three independent Authors (BR, RO, DB); disagreements were resolved by consensus.

For each of the included studies, a Microsoft Excel sheet was prepared for data extraction with the following information: first author, year of publication, active group(s) and comparator(s) (for RCTs), number of participants, number and proportion of women, rate(s) of response to triptans, definition of non-response to triptans, factors influencing response to triptans, previously failed triptans, number(s) and proportion(s) of triptan responders with previous triptan failures.

### Consensus development

The Consensus Panel included 16 Senior Members experienced in headache; 15 members were physicians - either neurologists or specialists in Internal Medicine -, while one was a pharmacologist. The Panel included three Junior Members (RO, BR, DB) who contributed to review of the literature and group discussion but did not vote.

As a first step, an online meeting was held to agree on the need for a new Consensus Statement and on the composition of the Consensus Panel and Junior Members. On that occasion, the Junior Members presented the results of the literature search. Thereafter, the process was carried out via e-mails and e-questionnaires and via web-meetings to have group discussions.

**Fig. 1:**
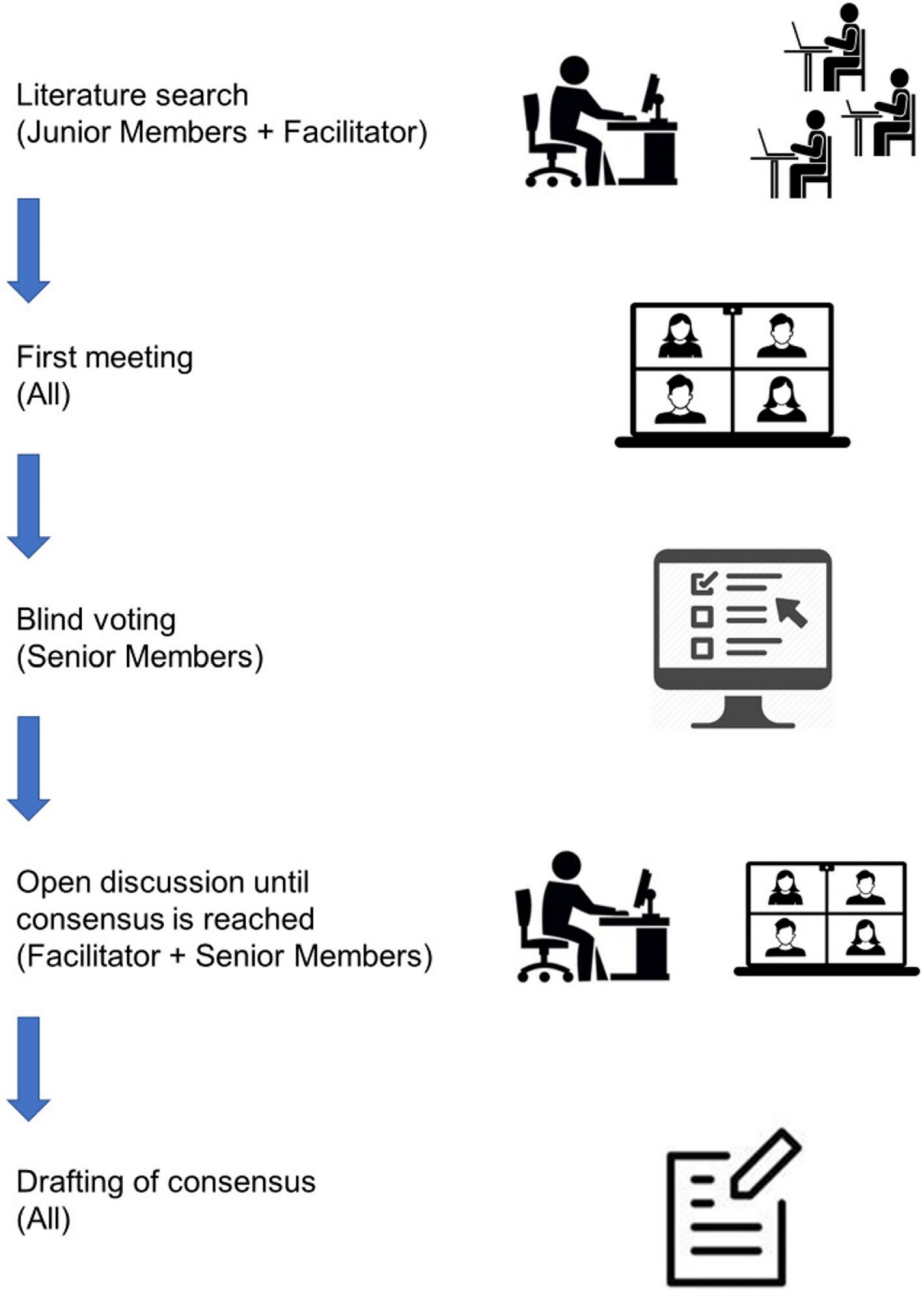
Consensus development process

To develop the consensus definition, we used a hybrid format including blind voting and opinion collection according to the Delphi method [21] and open group discussions (Fig. 1). The hybrid format was adopted to benefit from the advantages of both the Delphi method and open discussion.

In the first step, the Consensus Panel members were assigned to multiple-choice questions, with the option of adding text comments. Participants were instructed not to discuss responses among themselves at this stage. Considering responses to questions, a first consensus definition was drafted. This consensus definition was submitted to all the Panel members to collect additional comments. Comments were sent only to the facilitator (SS) who developed an updated definition. Thereafter, after a further revision of the definition to include additional suggestions, a web meeting was organized in order to have open discussion on the proposal. After the group discussion a second version of the proposal was drafted in and submitted to the entire group the get additional comments. At this stage, participants were again instructed not to discuss among themselves and to send their comments only to the facilitator. Thereafter, a final consensus version was drafted which was submitted to the panel for voting and approval by every group member.

REDCap data capture tools [22] hosted at the University of L’Aquila, Italy, were used to collect consensus responses.

## Results

### Systematic review

**Fig. 2:**
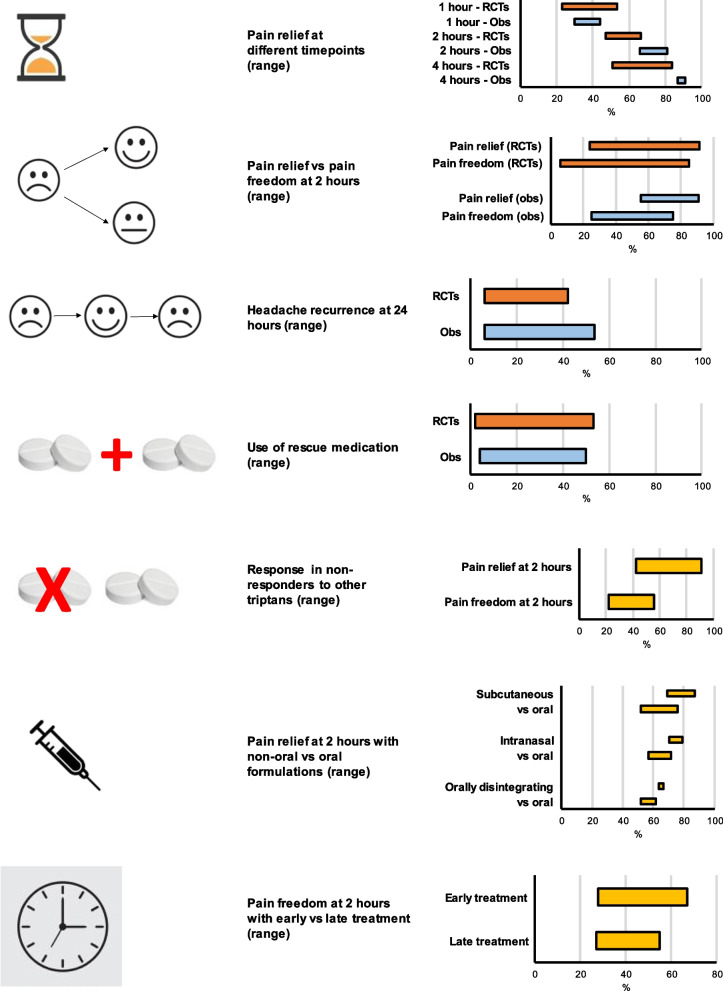
Summary of findings of the systematic review. Bars represent percentage ranges in the reviewed studies. Obs indicates observational studies; RCTs, randomized controlled trials

Database search retrieved 2824 results; after duplicate removal, screening of titles and abstracts, and full-text review, 251 records were left. Fig. S1 in Supplementary File reports the details of literature search. Supplementary Tables S1-S7 report detailed evidence of each included study, while an overall summary is reported in Fig. 2.

The results of the systematic review were summarized in seven categories:

1. *Pain relief at different time points*. RCTs: 23.0%–53.2% within 1 hour, 47.0–66.2% within 2 hours, 51–83.6% within 4 hours. Observational studies: 30% –44% within 1 hour, 66% –81% within 2 hours, 87%– 91% within 4 hours.
2. *Pain relief* versus *pain freedom within 2 hours from triptan administration*. RCTs: 23.9–91.2% pain relief, 6.0–85% pain freedom. Observational studies: 55.0–91.0% pain relief, 25.0–75.0% pain freedom.
3. *Headache recurrence within 24 hours from triptan administration*. RCTs: 6.0–42.0%. Observational studies: 6.0–53.4%
4. *Use of rescue medication*. RCTs: 1.8–53.1%. Observational studies: 3.9–50.0%.
5. *Response in non-responders to previous triptans*. Pain relief within 2 hours: 42.5–91.0%; pain freedom within 2 hours: 22.0–56.0%.
6. *Pain relief within 2 hours of consumption of non-oral* versus *oral triptan formulations*. Subcutaneous vs oral: 69.0–87.0% vs 52.0–76.0%. Intranasal vs oral: 70.3–79.2% vs 56.7–72.0%. Disintegrating tablets vs oral: 63.9–66.5% vs 52.0–61.6%.
7. *Pain freedom within 2 hours of early* versus *late treatment with triptans*. Pain freedom: 28.0–67.0% if treated early (variously defined as within 1 hour from onset, 4 hours from onset, or when the pain is mild) vs 27.0–55.0% if treated later.

### Consensus

The results of the multiple-choice questions, which were submitted in the first step of the consensus process are shown in Table S8.

**Fig. 3:**
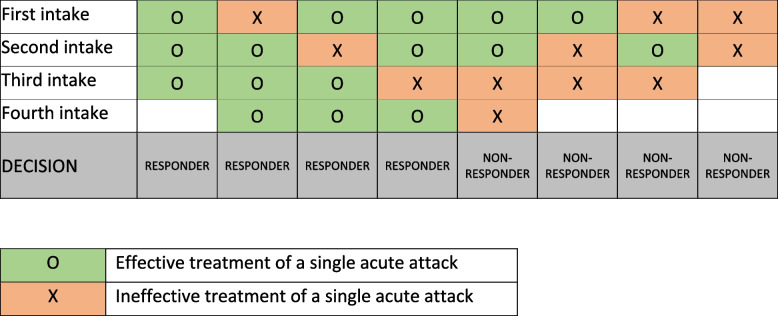
Decision aid to define a patient as responder or non-responder to a triptan based on the evaluation of the response to two-to-four consecutive attacks

**Fig. 4:**
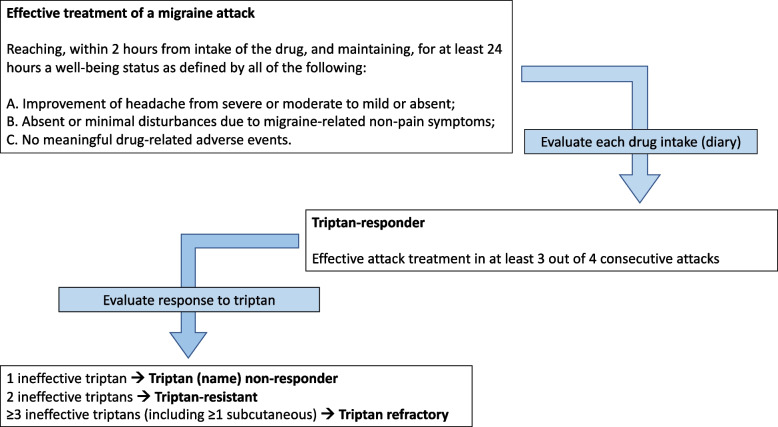
Consensus definitions of effective treatment, triptan-responder, and triptan non-responder

In the consensus we firstly provided a definition of *effective treatment of a migraine attack*, then provided a general definition of *triptan-responder*, and finally provided definitions of *triptan failure* (Figs. 3 and 4).

#### Definition of effective treatment of a migraine attack

The panel reached a consensus on the following definition of effective treatment of a migraine attack:

Reaching, within 2 h from intake of the drug, and maintaining, for at least 24 h a well-being status as defined by all the following:

A. Improvement of headache from severe or moderate to mild or absent;
B. Absent or minimal disturbances due to migraine-related non-pain symptoms;
C. No meaningful drug-related adverse events.

This definition is not specific for triptans but is applicable to any drug to treat an acute migraine attack, including combinations such as those of a triptan plus a non-steroidal anti-inflammatory drug. The Consensus Panel considered that a migraine attack is treated successfully if there is a “substantial improvement in pain”, there are “no relevant residual non-painful migraine related symptoms” and “no relevant adverse effects” from the drug. This definition acknowledges that migraine is not only pain but that there is a wide range of migraine-related non-painful symptoms. Some are common and are acknowledged as migraine most bothersome symptoms [23]. They include photophobia, nausea, and phonophobia. However, the Panel recognized that less common symptoms (e.g. cognitive disturbance) may also have a relevant impact on single individuals. Hence, a broader definition of “non-pain symptoms” was preferred over a precise description of symptoms. When a drug provides benefits on pain or other symptoms but leads to adverse events, which are relevant for the patient the attack is considered ineffectively treated. It is important to note that some patients may have early recurrence of migraine attacks despite an effective treatment.

#### Definition of triptan-responder

A given triptan leads to effective attack treatment in at least 3 out of 4 consecutive attacks. The individual with migraine is comfortable in planning activities because, if pain occurs, it can be controlled with the given triptan.

It is acknowledged that in some circumstances a triptan cannot lead to recovery of well-being, but the triptan may be considered effective in most attacks thus being associated with overall satisfaction with the drug. According to the Consensus Panel, an individual with migraine could be considered triptan-responder even if a rescue drug is needed in some attacks. The Consensus Panel agreed that not all attacks can be fully controlled; nevertheless, to be considered satisfactory, response to the drug should be consistent in the majority of the attacks. RCTs data showed consistent response to triptans across multiple attacks [24–32]. However, data could change in clinical practice. A proportion of at least 3 over 4 effectively treated attacks was considered acceptable by the Panel. When assessing the response to a triptan, it is also important to ensure prompt dosing, i.e. as close as possible to headache onset or when the pain is still mild (Table S7), as early treatment can enhance the effectiveness of acute drugs. Additionally, the responder status for a given triptan may change over time. This information cannot be captured from the literature as it would require very long times of observation; yet it is important from the perspective of the single individual.

#### Definition of triptan failure

Triptan failure includes cases where the condition of triptan-responder is not met. Considering the number of failed triptans, we propose the definitions reported below.

- *Triptan non-responder*: Failure of a single triptan (not matching the definition of triptan-responder)
- *Triptan-resistant*: Failure of at least 2 different triptans (each of them not matching the definition of triptan-responder).
- *Triptan refractory*: Failure to at least 3 different triptans, including subcutaneous formulation (each of them not matching the definition of drug-responder).
- *Triptan ineligibility*: Presence of an acknowledged contraindication to triptan use as reported in the summary of product characteristics (main contraindications which may vary across countries and drugs: coronary artery disease or angina, peripheral artery disease, stroke or TIA, severe renal and hepatic insufficiency).

As stated above, the assessment of effectiveness of a triptan should consider early administration. Individuals with migraine also need to be instructed to take at least a therapeutic dose. Although oral administration is usually easier than non-oral routes, gastric absorption of orally- administered medications can be delayed during migraine attacks in some individuals [10]. Alternative routes of administration, including parenteral, inhalational, buccal, intranasal, and rectal, can be utilized when rapid action is needed in difficult-to-treat attacks such as those with severe nausea or status migrainosus. The Consensus Panel agreed that subcutaneous formulations are generally more effective than oral formulations, as also reported in some trials (Fig. 2; Table S6 in Supplementary File). Although not deemed necessary to define resistance to triptans (Table S8 in Supplementary File), failure to subcutaneous triptans was considered mandatory for the definition of triptan refractoriness as subcutaneous formulations have the fastest absorption; thus, other triptan formulations with slower absorption are not expected to lead to triptan response. However, there are country-specific differences in the availability of triptan formulations, including the non-availability of subcutaneous sumatriptan in some countries. The Panel issued a broad statement that is independent from country-specific differences. However, this approach has the consequence that in some countries there are no individuals with migraine that can be defined as triptan non-responders.

The Panel deemed important to grade response to triptans in individuals with migraine to optimize acute treatment and identify individuals with unmet needs for acute treatment of migraine. Individuals who are triptan resistant or refractory are highly in need of novel drug classes to manage the acute attack; association of different drugs can also represent an option for those patients. Therefore, those individuals might be eligible for the upcoming acute treatments, which include ditans and gepants [33, 34]. From the point of view of basic science, defining a cohort of triptan-resistant or -refractory individuals could be important to define new pathophysiological mechanisms and pharmacological targets for the acute treatment of migraine.

The Consensus Panel identified criteria for ineligibility as well as for resistance or refractoriness to triptans. Ineligible individuals present comorbidities that are acknowledged contraindications to use a triptan, including a history of vascular events or severe renal and hepatic insufficiency. Treatment options different from triptans are advisable in those individuals. Those options might include non-steroidal anti-inflammatory drugs, acetaminophen, or novel upcoming drugs such as ditans and gepants.

## Discussion

The Consensus Panel proposed a definition of response to drugs for the acute treatment of migraine that is patient-centered, with the pivotal concept of patient reported “well-being”.

The initial intention of the Panel was to focus on response, resistance, and refractoriness to triptans; however, when considering each single drug, the same barriers to effective care are present for any drug class. The literature search on triptans faced a high heterogeneity in design, definition of outcomes, and results across the available RCTs and observational studies (Fig. 2). Therefore, most of the proposed definitions resulted from open discussion and personal experience of the Consensus Panel. The new definitions are primarily for use in clinical practice, to select individuals in need for the optimization of acute treatment. However, on the long run, these definitions can have a pathophysiological significance as the identification of group of individuals, resistant or refractory to triptans, could lead to the identification of new pharmacological targets.

When discussing the definitions, the consensus panel faced several issues, including the reporting of drug response or triptan non-response, the elements of variability in response or non-response to drugs, and the difference between resistance and refractoriness to triptans.

To establish efficacy of available treatments in clinic, it is important to use an attack report form (diary). An easy-to-use electronic or paper diary that captures predefined endpoints should be used. Data for acute attack treatment should be entered in real time to limit recall bias. Adverse events may also be collected in the diary. Reporting the degree of response to acute drugs could be important; however, we should be aware that complicated report forms with detailed description of symptoms may be difficult for subjects to fill out during attacks. The quantity and quality of collected data might be inversely proportional. Future clinical research should focus on the balance between a simple user friendly and an extensive complete report of attacks, by individuals with migraine. This applies not only to pain intensity, but also for non-pain symptoms, or adverse events, or the other components of well-being that could be impaired by migraine and restored by acute treatment.

As reported in Fig. 2, the proportion of response to triptans in terms of pain freedom or pain relief was extremely variable across studies. Studies available in the literature assessed some factors that potentially influence response to triptans, including the route and timing of administration. However, other factors, including the clinical presentation of migraine attacks, external triggers such as menstruation or stress, and pharmacogenomics should be further studied to obtain treatment optimization. The Consensus Panel agreed that not only pain, but also non-pain symptoms contribute to the satisfaction of individuals with migraine with their acute treatments. Considering the “most bothersome symptom” together with headache has become a mainstay of RCT for the acute treatment of migraine in recent years [35–38]. RCTs and observational studies on triptans were mostly focused on migraine pain without considering non-pain symptoms. Migraine-related disability and patient-reported outcomes were also poorly assessed by those studies and should receive more attention from physicians and researchers in the field of headache disorders. An important issue when considering response to drugs for the acute treatment of migraine is the duration of response, which includes the recurrence of pain and the need for rescue medication. It is unclear whether pain recurrence or the use of rescue medication impair the well-being of individuals with migraine or their satisfaction with use of triptans. The Consensus Panel did not reach a conclusion whether these parameters should be included in the definition of triptan response or non-response (Table S8). Pain recurrence and use of rescue medication should be included in the assessment of response to acute medication in clinical practice and undergo further testing before being considered into a definition of response or non-response.

A further issue worth considering is consistency of response to each triptan across multiple attacks. There is uncertainty about the number of attacks to be treated before declaring non-response to a triptan. The Consensus Panel suggested that a triptan could be considered effective if restoring well-being in at least three quarters of migraine attacks (Fig. 3). However, response to acute drugs is influenced by many factors, many of which are independent from the action of triptans, such as hormonal or psychological triggers. Monitoring response to triptans with a standardized tool such as a headache diary could help overcoming the barriers to an optimal acute treatment of migraine. Careful monitoring of that response might lead to the identification of novel strategies for optimization of acute migraine treatment. It could also lead to the rapid identification of individuals eligible to novel treatments or to experimental studies of acute treatments.

The present Consensus not only provided a definition referring to response to each single drug, but also referred to the individual with the introduction of “resistance” and “refractoriness” to triptans (Fig. 4). The cutoffs for resistance and refractoriness were arbitrary and mostly based on the Panel members’ clinical experience. Despite that, they could give an account of clinical conditions of different individuals. In individuals who are resistant to triptans, the drugs could still be used after optimization, while in those who are refractory the drugs should be abandoned in favor of other classes. The difference in response to the drugs could reflect genetically determined differences in the mechanisms and mediators of migraine attacks.

## Conclusions

Response and non-response to acute drugs for the treatment of migraine have been historically heterogeneously defined. The Consensus Panel provided experienced-based definitions that simplify the assessment of response to acute drugs and non-response to triptans in clinical practice. The new definitions help to identify suitable individuals for novel treatments and may support studies to gain insights into the pathophysiology of migraine attacks.

## Supplementary Information

**Additional file 1: Figure S1.** PRISMA flowchart of systematic review. **Table S1.** Pain relief at different timepoints. **Table S2.** Pain relief and pain-free at 2 hours. **Table S3.** Headache recurrence within 24 hours. **Table S4.** Rescue medication. **Table S5.** Response in triptan non-responders.** Table S6.** Comparisons of different triptan formulations. **Table S7.** Comparisons between early and late treatment with triptans. **Table S8.** Agreement on questions submitted in Round 1. **Table S9.** Conflicts of interest of the authors.

## Data Availability

There are no original data.

## Abbreviations

5-HT: 5-hydroxytriptamine (serotonin)
CGRP: Calcitonin gene-related peptide
PRISMA: Preferred Reporting Items for Systematic reviews and Meta-Analyses
