# Supplementary material for: European Headache Federation (EHF) consensus on the definition of effective treatment of a migraine attack and of triptan failure
Source: J Headache Pain. 2022 Oct 12;23(1):133. doi: 10.1186/s10194-022-01502-z (PMC9555163; doi:10.1186/s10194-022-01502-z)
Supplement: Supplementary file 1 — Additional file 1: Figure S1. PRISMA flowchart of systematic review. Table S1. Pain relief at different timepoints. Table S2. Pain relief and pain-free at 2 hours. Table S3. Headache recurrence within 24 hours. Table S4. Rescue medication. Table S5. Response in triptan non-responders. Table S6. Comparisons of different triptan formulations. Table S7. Comparisons between early and late treatment with triptans. Table S8. Agreement on questions submitted in Round 1. Table S9. Conflicts of interest of the authors. [file 10194_2022_1502_MOESM1_ESM.docx]

**Figure S1**. PRISMA flowchart of systematic review.

**
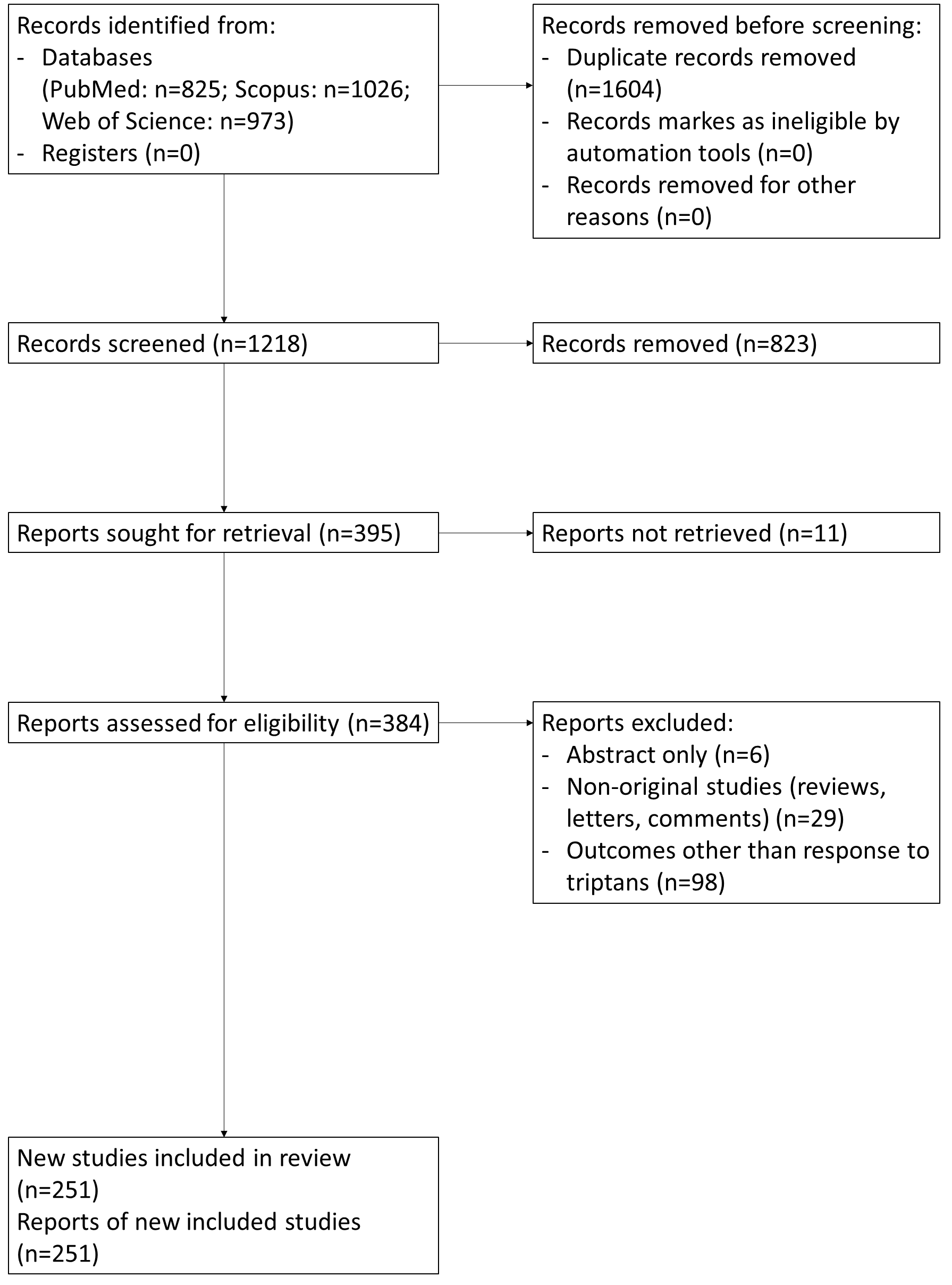
**

**Table S1**. Pain relief at different timepoints.

| **Reference** | **Design** | **Active group** | **Comparator (for RCTs)** | **Patients (n)** | **Women (n, %)** | **Pain relief at 1 hour, %** | **Pain relief at 2 hours, %** | **Pain relief at 4 hours, %** |
| --- | --- | --- | --- | --- | --- | --- | --- | --- |
| [1] | RCT | Zolmitriptan 5 mg nasal spray | Placebo | 1868 | 85.1-88.3% | 53.2 | 66.2 | 72.9 |
| [2] | RCT | Zolmitriptan 2.5 mg ODT | Placebo | 471 | 86-88% | 45 | 63 | 51 |
| [3] | RCT | Zolmitriptan 2.5 or 5 mg | Sumatriptan 25 or 50 mg | 1212 | 84,4-89,8% | 35 (2.5 mg), 37.4 (5 mg), 32.9 (suma 25 mg), 34.7 (suma 50 mg) | 67.1 (2.5 mg), 64.8 (5 mg) vs 65.9 (suma 25 mg) and 63.8 (suma 50 mg) | 83.3 (2.5 mg), 83.6 (5 mg) vs 75.8 (suma 25 mg) and 80.8 (suma 50 mg) |
| [4] | RCT | Zolmitriptan 1mg, 2m.5 mg, 5 mg, 10 mg | Placebo | 999 | 876 (86) | 36-51 | 53-67 | 57-78 |
| [5] | RCT | Zolmitriptan 2.5 mg /5 mg | Placebo | 734 | 628 (85.6) | 40.1 (2.5 mg), 40.0 (5 mg) | 58.9 (2.5 mg), 62.1 (5 mg) | 71.3 (2.5 mg), 74.3 (5 mg) |
| [6] | RCT | Eletriptan 20 mg, 40 mg, 80 mg oral | Placebo | 1190 | 85-90% | 24 (20 mg), 34 (40 mg), and 32 (80 mg) | 47 (20 mg), 62 (40 mg), 59 (80 mg) | 64 (20 mg), 76 (40 mg), 79 (80 mg) |
| [7] | RCT | sumatriptan 50 mg oral | naproxen sodium 500 mg, combination of sumatriptan 50 mg and naproxen sodium 500 mg, or placebo | 972 | 880, 90,5% | 23 | 49 | 56 |
| [8] | RCT | Zolmitriptan oral 2.5 mg | Placebo | 334 | 100% | 40.6 | 65.7 | 81.7 |
| [9] | Open-label, single-arm study | Eletriptan 40 mg p.o. | - | 113 | 81% | 30 | 66 | 87 |
| [10] | Open-label, single-arm study | Eletriptan 40 mg p.o. | - | 110 | 75% | 44 | 81 | 91 |

**Table S2**. Pain relief and pain-free at 2 hours.

| **Reference** | **Design** | **Active drug** | **Comparator (for RCTs)** | **Patients (n)** | **Women (n, %)** | **2-hour pain relief, %** | **2-hour pain-free, %** |
| --- | --- | --- | --- | --- | --- | --- | --- |
| [11] | RCT | Sumatriptan 100 mg ODT | Placebo | 233 | 198 (85%) | 50 | 26 |
| [12] | RCT | Eletriptan 20/40/80 mg p.o. | Placebo | 321 | 238 (74%) | 64 (20 mg), 67 (40 mg), 76 (80 mg) | 24 (20 mg), 22 (40 mg), 28 (80 mg) |
| [13] | RCT | Eletriptan 40/80 mg | Placebo | 971 | 83% | 77 in 2 or more out of 3 attacks with eletriptan 40 mg, 73 with eletriptan 80 mg; 46 in 3/3 attacks with eletriptan 40 mg, 47 with eletriptan 80 mg | 42 (ELE-40), 38 (ELE-80); in 3/3 attacks: 18 (ELE-40), 17 (ELE-80). |
| [14] | RCT | Sumatriptan 50 mg + Promethazine | Sumatriptan 50 mg + Placebo | 242 | 74.3% | 62.2 with sumatriptan/promethazine, 37.2 with sumatriptan | 39.6 with sumatriptan+promethazine, 26.3 with sumatriptan |
| [15] | RCT | Rizatriptan 10 mg p.o. / Dexamethasone 4 mg | Rizatriptan 10 mg p.o. | 35 | 100% | 71.1 (rizatriptan), ~88 (combination) | 50.8 (rizatriptan), ~58 (combination) |
| [16] | RCT | Sumatriptan iontophoretic transdermic system 6.5 mg | Placebo | 454 | 389 (84.9%) | 52.9 | 18.0 |
| [17] | RCT | Sumatriptan 85 mg + naproxen 500 mg | Sumatriptan 85 mg/ Naproxen 500 mg/ Placebo | 3413 | 88% | 57-65 with sumatriptan + naproxen, 50-55 with sumatriptan monotherapy | 30-34 (suma+naproxen), 23-25 (sumatriptan monotherapy) |
| [18] | RCT | Sumatriptan 6 mg s.c. | Placebo | 200 | 165 (83%) | 66-88 in Non-Caucasians (Black, Hispanic, and other), 78-93 in Caucasians | 45-65 in Non-Caucasians, 55-79 in Caucasians |
| [19] | RCT | Almotriptan 2/6/10 mg s.c. | Placebo | 123 | 100 (81.3%) | 96.5 with almotriptan 6 mg, 90.3 with almotriptan 10 mg, ~60 with 2 mg | 58.6 with almotriptan 6 mg, 38.7 with almotriptan 10 mg, 25.8 with 2 mg |
| [20] | RCT | Sumatriptan 100 mg p.o. | Placebo | 3706 | 83-86% | 57 | 30 |
| [21] | RCT | Zolmitriptan 5/10/15/20 mg p.o. | Placebo | 840 | 76-86% | 66, 71, 69 and 77 for 5 mg, 10 mg, 15 mg and 20 mg, respectively | 39, 39, 43 and 47 for 5 mg, 10 mg, 15 mg and 20 mg, respectively |
| [22] | RCT | Almotriptan 2/6.25/12.5/25 mg p.o. | Placebo | 742 | 85% | 30 (2.5 mg), 56.3 (6.25 mg), 58.5 (12.5 mg), 66.5 (25 mg) | 17.1 (2mg), 37.8 (12.5 mg), 45.3 (25 mg) |
| [23] | RCT | Rizatriptan 10 mg p.o. | Placebo | 407 | 341 (84%) | 86 | 48 |
| [24] | RCT | Almotriptan 12.5 mg p.o. | Placebo | 404 | 84% | in patients with allodynia: 53 and without allodynia: 72 | with allodynia: 31.4 and without allodynia: 44.3 |
| [25] | RCT | Sumatriptan 25 mg supp. | Indomethacin 25 mg /Prochlorperazine 4 mg/ Caffeine 75 mg supp. | 112 | 72% | 65 | 34 |
| [26] | RCT | Almotriptan 12.5 mg p.o. | Placebo | 275 | 100% | 77.4 for menstrual-related migraine (MRM) and 68.3 for non-MRM | 35.4 for MRM and 35.9 for non-MRM |
| [27] | RCT | Sumatriptan 6 mg s.c. | 1.8 g lysine acetylsalicylate iv/ placebo | 278 | 220 (80%) | 91.2 | 76.3 |
| [28] | RCT | Almotriptan 12.5 mg p.o. | Placebo | 198 | 85.8-88.0% | 50 (moderate pain), 46.4 (severe pain) | 36.7 (moderate pain), 31.9 (severre pain) |
| [29] | RCT | Almotriptan 12.5 mg p.o. | Placebo | 221 | 85.8-88% | 47.5 | 33.3 |
| [30] | RCT | Eletriptan 40/80 mg p.o. | Cafergot (1 mg ergotamine tartrate + 100 mg caffeine) | 733 | 86-90% | 68 with eletriptan 80 mg and 54 with eletriptan 40 mg | 38 with 80 mg, 28 with 40 mg |
| [31] | RCT | Sumatriptan 6 mg s.c. | Alniditan 1.4/2.8 mg or placebo | 924 | 86.4% | 87.1 | 65.9 |
| [1] | RCT | Zolmitriptan 5 mg nasal spray | Placebo | 1868 | 85.1-88.3% | 66.2 | 35.6 |
| [32] | RCT | Zolmitriptan 5/2.5/1/0.5 mg nasal spray | Placebo | 1093 | 82.2% | 73.2, 70.5, 49.9 and 41.5 for 5, 2.5, 1 and 0.5 mg respectively | 51.5, 48.1, 24.7 and 21.8 for 5, 2.5, 1 and 0.5 mg respectively |
| [2] | RCT | Zolmitriptan 2.5 mg ODT | Placebo | 471 | 86-88% | 63 | 27 |
| [33] | RCT | Almotriptan 12.5/25 mg p.o. | Sumatriptan 100 mg / Placebo | 668 | 83.2-88.9% | 56.8 for almotriptan 12.5 mg, 56.5 for almotriptan 25 mg and 63.7 for sumatriptan | pts with moderate pain: 38.7 (almo12.5), 46.7 (almo25), 36.9 (suma100); with severe pain: 16.7 (almo12.5), 19.8 (almo 25), 29.3 (suma100) |
| [34] | RCT | Sumatriptan 6 mg s.c. | Placebo | 179 | 100% | 73 | 55 |
| [35] | RCT | Eletriptan 40/80 mg | Placebo | 446 | 85-91% | 59 for 40 mg, 70 for 80 mg | 35 for 40 mg, 42 for 80 mg |
| [36] | RCT | Frovatriptan 2.5 mg | Rizatriptan 10 mg (study 1), zolmitriptan 2.5 mg (study 2), almotriptan 12.5 mg (study 3) | 346 | 81% | in men: 56 frovatriptan vs. 57 comparators; in women: 55 vs. 57 . | Men: 32 with frovatriptan, 38 with other triptans. Women: 30 with frovatriptan, 33 with other triptans |
| [12] | RCT | eletriptan 20 mg, 40 mg and 80 mg | Placebo | 312 | 68-79% | 64 (20 mg), 67 (40 mg), 76 (80 mg) | 24 (20 mg), 22 (40 mg), 28 (80 mg) |
| [37] | RCT | Eletriptan 40 mg | Naratriptan 2.5 mg or placebo | 483 | 79-82% | 56 (suma), 42 (nara) | 35 (suma) 18 (nara) |
| [38] | RCT | Zolmitriptan 5 mg nasal spray | Placebo | 912 | 86.3-88.7% | 23.9 | 35.7 |
| [39] | RCT | Zolmitriptan 2.5 mg | Combination of Acetylsalicylic Acid and Metoclopramide | 719 | 83.7-86.2% | 33.4 (3 attacks) | 10.7 (3 attacks) |
| [40] | RCT | eletriptan 20 mg, 40 mg and 80 mg | Sumatriptan 100 mg or placebo | 692 | 80-85% | 54 for eletriptan 20 mg; 65 for eletriptan 40 mg; 77 for eletriptan 80 mg; 55 for sumatriptan 100 mg | 19, 29, 37, 23, respectively |
| [41] | RCT | Rizatriptan 5 and 10 mg | Sumatriptan 25 and 50 mg or placebo | 1329 | 88-91% | 68 (riza 5 mg), 72 (riza 10 mg), 62 (suma 25 mg), 68 (suma 50 mg). | 33 (riza 5 mg), 41 (riza 10 mg), 28 (suma 25 mg), 37 (suma 50 mg) |
| [42] | RCT | Sumatriptan 50 mg oral | Placebo | 23 | 12, 52% | 30.4 | 21.7 |
| [43] | RCT | Rizatriptan 5 or 10 mg oral | Placebo | 1382 (997 received medication) | 550 (56.3%) | 58.8 | 30.6 |
| [44] | RCT | sumatriptan 100 mg tablets | Placebo | 253 | 184 (72.7) | 49 | 25 |
| [45] | RCT | Zolmitriptan nasal spray | Placebo (single-blind placebo challenge) | 248 | 98 (57.3) | 51.4 | 39.2 |
| [46] | RCT | Sumatriptan 50 mg | Placebo | 249 | (86) | 47 (migraine), 53 (migrainous headache), 63 (tension-type headache) | 18 (migraine), 26 (migrainous headache), 28 (tension-type headache) |
| [47] | RCT | Zolmitriptan 10 mg orally disintegrating tablet | Sumatriptan 50 mg | 524 | 429 (82.0) | 84 (riza), 81 (suma) | 60 (riza), 52 (suma) |
| [48] | RCT | Zolmitriptan 5 mg | Rizatriptan 10 mg | 48 | NR | 73 (zolmi), 81 (riza) | 45 (zolmi), 58 (riza) |
| [49] | RCT | Zolmitriptan 2.5 mg / 5 mg | Placebo | 2800 (Part 1), 2499 (Part 2) | 2399 (85.7), 2152 (86.1) | 80 (5 mg), 73 (2.5 mg) | 64 (5 mg), 52 (2.5 mg) |
| [11] | RCT | Sumatriptan 100 mg | Placebo | 233 | 198 (85.0) | 50 | 26 |
| [50] | RCT | Frovatriptan 2.5 mg | Placebo | 229 | 207 (90.4) | 52.9 | 19.’ |
| [51] | RCT | Almotriptan 6.25 mg / 12.5 mg | Placebo | 909 | 788 (86.7) | 60 (6.25 mg), 70 (12.5 mg) (at least 1/3 attacks) | 21.9, 20.4 (at least 1/3 attacks) |
| [52] | RCT | Sumatriptan nasal spray (2.5 mg, 5 mg, 10 mg, 20 mg) | Placebo | 544 | 461 (84.7) | 49 (5 mg), 46 (10 mg), 64 (20 mg) | 42 (20 mg), 14-24 (2.5-10 mg) |
| [53] | RCT | Ketorolac 31.5 mg nasal spray / sumatriptan 20 mg nasal spray | Placebo | 54 | 53 (98.1) | 72.5 (ketorolac), 69.4 (sumatriptan) | 43.1, 36.7 |
| [54] | RCT | Frovatriptan 0.5 to 40 mg | Placebo | 1453 | 1249 (86.0) | 38-48 | 14-21 |
| [4] | RCT | Zolmitriptan 1mg, 2m.5 mg, 5 mg, 10 mg | Placebo | 999 | 876 (86) | 53-67 | 16-36 |
| [55] | RCT | Zolmitriptan 2.5 mg / 5 mg / 10 mg | Placebo | 850 | 408 (58.6) | 53-57 | 19-25 |
| [56] | RCT | Frovatriptan 2.5 mg | Placebo | 2194 | 1915 (87.3) | 37-46 | 9-14 |
| [57] | RCT | Eletriptan 20 mg /40 mg /80 mg | Placebo | 563 (Europe), 321 (Japan), 1190 (USA) | 74% to 87% | 47-77 | 14-37 |
| [58] | RCT | Zolmitriptan 1,2.5,5 mg | Placebo | 202 | 150 (74.3) | 53.5-65.4 | 17.8-23.1 |
| [59] | RCT | Eletriptan 40 mg / 80 mg | Sumatriptan 50 mg / 100 mg | 774 | NR | 50-67 | 18-31 |
| [60] | RCT | Rizatriptan 10 mg oral disintegrating tablets | Placebo | 100 | 92 (92.0) | 55 | 36 |
| [61] | RCT | Rizatriptan 10 mg oral disintegrating tablets | Placebo | 102 | 88 (86.3) | 51 | 22 |
| [6] | RCT | Eletriptan 20 mg, 40 mg, 80 mg oral | Placebo | 1190 | 85-90% | 47 (20 mg), 62(40 mg), 59 (80 mg) | 14, 27, and 27 in the 20-mg, 40-mg, and 80-mg groups |
| [62] | RCT | Sumatriptan Fast-Disintegrating/Rapid-Release Formulation tablets 50 mg or 100 mg | Placebo | 3331 | 84-88% | 67 (50 mg), 72 (100 mg) | 40 (50 mg), 47 (100 mg) |
| [7] | RCT | sumatriptan 50 mg oral | naproxen sodium 500 mg, combination of sumatriptan 50 mg and naproxen sodium 500 mg, or placebo | 972 | 880, 90,5% | 49 | 20 |
| [63] | RCT | Zolmitriptan 2.5 mg | Placebo | 301 | 276, 85% | 62 | 22 |
| [64] | RCT | Zolmitriptan 5 mg ODT | Placebo | 670 | 85,4-87,7% | 49 | 31.1 |
| [65] | RCT | Naratriptan 2.5 mg oral | Sumatriptan 50 mg oral or placebo | 347 | 90% | 25 | 6 |
| [66] | RCT | Eletriptan 40 and 80 mg | Zolmitriptan 2.5 mg or placebo | 1312 | 83-88% | 74 (ele 80 mg), 64 (ele 40 mg), 60 (zolmi) | 44 (ele 80 mg), 32 (ele 40 mg), 26 (zolmi) |
| [67] | RCT | Eletriptan 40, or 80 mg | Rizatriptan 10 mg | 79 | 78.5% | 88, 81, and 62 of patients in the eletriptan 40-mg, eletriptan 80-mg, and rizatriptan 10-mg groups | 77, 85, and 59 |
| [68] | RCT | Sumatriptan intranasal 22 mg | Sumatriptan oral 100 mg | 262 | 222, 84.7% | 79.6 (intranasal), 76.9 (oral) | 60.4 (nose), 56.3 (oral). |
| [69] | RCT | Zolmitriptan 2.5 mg (tablet A), followed by 2.5 or 5 mg zolmitriptan (tablet B) | Zolmitriptan 2.5 mg (tablet A), followed after 2h by placebo | 2793 | 2399/2800 (85.7%) | 62.2 (Tablet A), 51.6 (solmi 5 mg), 49.7 (zolmi 2.5 mg) | 32.5 (Tablet A), 36.0 (solmi 5 mg), 31.0 (zolmi 2.5 mg) |
| [70] | RCT | Rizatriptan oral 5 or 10 mg | Sumatriptan oral 100 mg; placebo | 1099 | 1030 (81%) | 60 (riza5), 67 (RIZA10), 62 (SUMA) | 25 (riza5), 40 (riza10), 33 (suma) |
| [11] | RCT | Sumatriptan 100 mg | Placebo | 232 | 83-86% | 50 | 26 |
| [71] | RCT | frovatriptan plus dexketoprofen (25 mg or 37.5 mg) | frovatriptan 2.5 mg | 279 | 248 (89%) | 64 (frova), 84 (FroDex25), 80 (FroDex37.5) | 29 (27/93) with Frova, 51 (48/95) for FroDex25 and 51 (46/91) for FroDex37.5 |
| [72] | RCT | zolmitriptan 2.5 mg, rizatriptan 10 mg, sumatriptan 100 mg, almotriptan 12.5 mg, and eletriptan 40 mg | zolmitriptan 2.5 mg, rizatriptan 10 mg, sumatriptan 100 mg, almotriptan 12.5 mg, and eletriptan 40 mg (within-patient comparison) | 30 | 18 (60%) | 84.7 (zolmi), 81.3 (riza), 74.7 (almo), ~75 (ele), ˜80 (suma) | Pain-free at 2h: 66.0 (riza), 63.3 (ele), suma (50), 54.0 (almo), 54.7 (zolmi) |
| [73] | RCT | Sumatriptan intranasal 20 mg | Placebo | 56 | 48 (85.7%) | 60 | 33 |
| [74] | RCT | Zolmitriptan nasal spray 0.5, 2.5, or 5 mg | Placebo | 798 | 493 (61.8%) | 51 | 30 |
| [75] | RCT | Rizatriptan 5 mg | Placebo | 296 | 54% | 66 | 32 |
| [76] | RCT | Eletriptan 40 mg | Placebo | 274 | 56-59% | 57 | 22 |
| [77] | Longitudinal, long-term | Zolmitriptan 5 mg oral | - | 2058 | 1769 (86%) | 81 | 55 |
| [78] | Prospective, open-label, pilot cohort study | Frovatriptan 2.5 mg | - | 20 | 100% | 55 | 10 |
| [79] | Open-label study | Sumatriptan 50 mg oral | - | 72 | 56 (77.8%) | 81.9 | 61.1 |
| [80] | Open-label, single-arm, prospective study | Triptans (naratriptan, sumatriptan, rizatriptan, zolmitriptan) under preventive treatment with topiramate | - | 21 | 20 (95.2%) | : 80.3 before topiramate treatment, 82.2 under topiramate treatment | 46.9 before topiramate treatment, 44.6 under topiramate treatment |
| [81] | Long-term, open-label, single-arm, prospective study | Almotriptan 12.5 mg p.o. | - | 420 | 233 (55.5%) | 61.7 | 40.5 |
| [77] | Longitudinal, long-term | Zolmitriptan 5 mg | - | 2058 | 86% | 81 | 55 |
| [82] | Open-label, single-arm, prospective study | Sumatriptan 6 mg s.c. | - | PP: 212; safety: 242 | PP: 82.5%; safety: 81.0% | 85.9 | 60.7 |
| [9] | Open-label, single-arm study | Eletriptan 40 mg p.o. | - | 113 | 81% | 66 | 25 |
| [10] | Open-label, single-arm study | Eletriptan 40 mg p.o. | - | 110 | 75% | 81 | 75 |
| [83] | Open-label, single-arm study | Sumatriptan 6 mg s.c. | - | 43 | - | 91 | 56 |
| [84] | Open-label, single-arm study | Eletriptan 40 mg | - | 123 | 80% | 63.8 | 30.2 |
| [85] | Real-world study investigating headache return | Triptans (sumatriptan, rizatriptan, eletriptan, zolmitriptan, almotriptan, sumatriptan injections), triptan nasal sprays | - | 411 | 91% | 66.2 | 34.1 |
| [69] | Open-label, long-term extension study | Zolmitriptan 2.5 mg | - | 2499 | 86% | 72.0 | 46.8 |
| [86] | Open-label, long-term study | Zolmitriptan 2.5 or 5 mg | - | 2499 | 2152 (86.1%) | 85 (2.5 mg), 79 (5 mg) | 69 (2.5 mg), 59 (5 mg) |
| [87] | Real-life, open-label, non-comparative outpatient study | Eletriptan | - | 611 | 86.2% | 87.3 | 50.6 |
| [88] | Prospective (open-label extension of RCT) | Sumatriptan 3mg sc | - | 234 | (85.4) | 83.4, 88.4, 84.1, 81.7 in attacks 1,2,3, and 4, respectively | 57.6, 64.6, 61.6, 66.3 in attacks 1, 2, 3, and 4, respectively |
| [89] | Prospective | Sumatriptan 6 mg sc auto-injector | - | 63 | 51 (81.0) | 93.7 | 60.3 |
| [90] | Open-label | Eletriptan 40 mg as a switch from butalbital-containing medications | - | 160 | (85) | 71 | 37 |
| [91] | Prospective | Eletriptan 40 mg | - | 437 | (85) | 62 | 36 |
| [92] | Open-label | Sumatriptan 5,10,20 mg | - | 437 | (53) | 71-85 | 40.52 |
| [93] | Open-label | Sumatriptan sc (SUMAVEL) | - | 90 | (83.3) | 81.6 | 48.0 |

**Table S3**. Headache recurrence within 24 hours.

| **Study number** | **Design** | **Active drug** | **Comparator** | **Patients (n)** | **Women (n, %)** | **Headache recurrence. %** |
| --- | --- | --- | --- | --- | --- | --- |
| [94] | RCT | Sumatriptan Succinate 25 mg + 25 mg | Sumatriptan Succinate Placebo; Combination of Isometheptene Mucate 65 mg, Dichloralphenazone 100 mg, and Acetaminophen 325; Isometheptene Combination Placebo | 126 | 86.1-91.8% | 11 (isometheptene), 10 (suma) |
| [37] | RCT | Eletriptan 40 mg | Naratriptan 2.5 mg or placebo | 483 | 79-82% | 29 (suma), 26 (nara) |
| [95] | RCT | Zolmatriptan 5 mg | Sumatriptan 100 mg or placebo | 1311 | 83-86% | 26 (zolmi), 28 (suma) |
| [96] | RCT | Almotriptan 12.5 mg | Placebo | 403 | 84% | 6 (mild/early), 24 (moderate/severe) |
| [97] | RCT | Sumatriptan 100 mg | Placebo | 61 | NR | 39 |
| [98] | RCT | Frovatriptan 2.5, 5, 10, 20, and 40 mg | Placebo | 635 | 508~~,~~ (73.1%) | 6-21 |
| [99] | RCT | Rizatriptan 10 mg (open-label) | Rizatriptan 10 mg + rofecoxib 25 mg (open-label)  Rizatriptan 10 mg + tolfenamic acid 100 mg (open-label) | 45 | 35 (78%) | 50.0 (rizatriptan) vs 15.4 (rizatriptan+rofecoxib) vs 20.3 (rizatriptan+tolfenamic acid) |
| [100] | RCT | Rizatriptan 10 mg + trimebutine 200 mg | Rizatriptan 10 mg | 32 | 24 (75%) | 31.2 |
| [47] | RCT | Zolmitriptan 10 mg orally disintegrating tablet | Sumatriptan 50 mg | 524 | 429 (82.0%) | 28 (riza), 30 (suma) |
| [101] | RCT | Eletriptan 40 mg or sumatriptan 100 mg | Placebo | 2113 | 86-87% | 31 (ele), 37 (suma) |
| [102] | RCT | Sumatriptan 100 mg | Placebo | 233 | 198 (85.0%) | 42 |
| [50] | RCT | Frovatriptan 2.5 mg | Placebo | 229 | 207 (90.4%) | 16.1 |
| [52] | RCT | Sumatriptan nasal spray (2.5 mg, 5 mg, 10 mg, 20 mg) | Placebo | 544 | 461 (84.7%) | 30-41 |
| [54] | RCT | Frovatriptan 0.5 to 40 mg | Placebo | 1453 | 1249 (86.0%) | 9-16 |
| [4] | RCT | Zolmitriptan 1 mg, 2~~m~~.5 mg, 5 mg, 10 mg | Placebo | 999 | 876 (86%) | 26-36 |
| [5] | RCT | Zolmitriptan 2.5 mg /5 mg | Placebo | 734 | 628 (85.6%) | 33.2 (2.5 mg), 30.5 (5 mg) |
| [56] | RCT | Frovatriptan 2.5 mg | Placebo | 2194 | 1915 (87.3%~~)~~ | 10-25 |
| [57] | RCT | Eletriptan 20 mg /40 mg /80 mg | Placebo | 563 (Europe), 321 (Japan), 1190 (USA) | 74-~~% to~~ 87% | 21-53 |
| [58] | RCT | Zolmitriptan 1, 2.5, 5 mg | Placebo | 202 | 150 (74.3%) | 15.7-28.1 |
| [59] | RCT | Eletriptan 40 mg / 80 mg | Sumatriptan 50 mg / 100 mg | 774 | NR | 16-27 |
| [103] | RCT | Rizatriptan 2.5 mg / 5 mg / 10 mg | Placebo | 249 | 222 (89%) | 39 |
| [104] | RCT | Sumatriptan 100 mg | Placebo | 1440 | 1226 (85.1%) | 25-29 |
| [6] | RCT | Eletriptan 20 mg, 40 mg, 80 mg oral | Placebo | 1190 | 85-90% | 31, 29, and 21 in the 20-mg, 40-mg, and 80-mg groups |
| [7] | RCT | sumatriptan 50 mg oral | naproxen sodium 500 mg, combination of sumatriptan 50 mg and naproxen sodium 500 mg, or placebo | 972 | 880~~,~~ (90,5%) | 41 |
| [63] | RCT | Zolmitriptan 2.5 mg | Placebo | 301 | 276~~,~~ (85%) | 22 |
| [102] | RCT | Sumatriptan 100 mg | Placebo | 232 | 83-86% | 42 |
| [73] | RCT | Sumatriptan intranasal 20 mg | Placebo | 56 | 48 (85.7%) | 19 |
| [75] | RCT | Rizatriptan 5 mg | Placebo | 296 | 54% | 11 |
| [76] | RCT | Eletriptan 40 mg | Placebo | 274 | 56-59% | 9 |
| [105] | Prospective observational study | frovatriptan 2.5 mg | - | 1620 | 1227~~,~~ (80.8%) | 11.24 (frovatriptan taken at low symptom severity), 13.97 (frovatriptan taken at high severity) |
| [84] | Open-label, single-arm study | Eletriptan 40 mg | - | 123 | 80% | 36.6 |
| [85] | Real-world study investigating headache return | Triptans (sumatriptan, rizatriptan, eletriptan, zolmitriptan, almotriptan, sumatriptan injections), triptan nasal sprays | - | 411 | 91% | 53.4 |
| [106] | Long-term, open-label study | Frovatriptan 2.5-7.5 mg oral | - | 486 | 438 (88.3%) | 6 |
| [107] | Prospective | Frovatriptan | - | 16737 | 13550 (81.0%) | 29.0 (long duration migraine), 12.6 (short duration migraine) |
| [108] | Pharmacogenetic study | Sumatriptan sc | - | 40 | 35 (87.5%) | 30 |
| [109] | Pharmacogenetic study | Sumatriptan sc | - | 40 | 35 (87.5%) | 30 |
| [110] | Cross-sectional survey | Any triptan | - | 109 | 89 (82%) | 14.3 |
| [111] | Prospective | Almotriptan 12.5 mg | - | 582 | (84.4%) | 12.9 if pain treated when mild, 25.0 if treated when moderate-to-severe |
| [112] | Pharmacogenetic study | Sumatriptan sc | - | 40 | 35 (87.5%) | 30 |
| [92] | Open-label | Sumatriptan 5, 10, 20 mg | - | 437 | (53%) | 7-15 |
| [113] | Open-label | Sumatriptan sc | - | 479 | 401 (84%) | 31 |
| [114] | Open-label | Eletriptan 80 mg vs sumatriptan 6 mg sc | - | 311 | (81%) | 25 (ele), 40 (suma) |

**Table S4**. Rescue medication

| **Study number** | **Design** | **Active drug** | **Comparator** | **Patients (n)** | **Women (n, %)** | **Rescue medication, %** |
| --- | --- | --- | --- | --- | --- | --- |
| [115] | RCT | Rizatriptan 5/10 mg oral | Placebo | 96 | 63 (66%) | 18% (n=17/96) and 22% (n=21/96) of the patients after the first and the second dose of rizatriptan. |
| [14] | RCT | Sumatriptan 50 mg + Promethazine | Sumatriptan 50 mg + Placebo | 242 | 74.3% | At 4h post-dose: 13.2% (sumatriptan+ placebo) and 24.5% (sumatriptan+Promethazine) |
| [116] | RCT | Sumatriptan 200 mg p.o. | Placebo | 94 | 85% | 27 |
| [117] | RCT | Rizatriptan 10 mg p.o. | Placebo | 80 | 67 (83.8%) | 15 |
| [118] | RCT | Frovatriptan 2.5 mg p.o. | Placebo | 275 | 239 (86.9%) | Second dose of study medication or rescue medication in the 24 h following early use of frovatriptan: 50%; later use: 68%. In the early use group, 80% of patients did not use rescue medication in the 24 h after dosing. |
| [119] | RCT | Almotriptan 12.5 mg p.o. | Placebo | 1767 | NR | In patients with pretreatment mild or moderate pain in the absence of allodynia-associated symptoms (AAS): 36.1%; with severe pain in the presence of AAS: 46.7%.  In the almotriptan-treated AEGIS population, 30.8% vs 56.0% in the ET 1 hour AIMS population, 9.7% vs 34.2% in the ST 1 hour AIMS population, and 45.2% vs 64.2% in the ST > 1 hour AIMS population. |
| [120] | RCT | Rizatriptan 10 mg ODT | Placebo | 188 | 90% | Up to 24h: 34% |
| [121] | RCT | Sumatriptan 8 mg s.c. | Placebo | 27 | 22 (81.5%) | 11 |
| [24] | RCT | Almotriptan 12.5 mg p.o. | Placebo | 404 | 84% | In pts with allodynia who treated when pain was mild: 15% vs those delaying: 45%; those without allodynia: 29% vs 23%. |
| [25] | RCT | Sumatriptan 25 mg supp. | Indomethacin 25 mg /Prochlorperazine 4 mg/ Caffeine 75 mg supp. | 112 | 72% | 12 |
| [27] | RCT | Sumatriptan 6 mg s.c. | 1.8 g lysine acetylsalicylate iv/ placebo | 278 | 220 (80%) | 1.8 |
| [29] | RCT | Almotriptan 12.5 mg p.o. | Placebo | 221 | 85.8-88% | 26.6 |
| [1] | RCT | Zolmitriptan 5 mg nasal spray | Placebo | 1868 | 85.1-88.3% | 30.8 |
| [122] | RCT | Sumatriptan 1.3 mg/1.8 mg s.c. | Placebo | 519 | NR | 38 (6 mg), 35 (8 mg) |
| [34] | RCT | Sumatriptan 6 mg s.c. | Placebo | 179 | 100% | 24% at 2h |
| [123] | RCT | Rizatriptan 10 mg ODT | Placebo | 297 | 89.5-89.7% | At 24h: 27% |
| [124] | RCT | Almotriptan 12.5 mg | early treatment (ET) vs standard treatment (ST) | 1304 | 89.1-85.5% | 35.5 (ET) vs 36.9 (ST) |
| [12] | RCT | eletriptan 20 mg, 40 mg and 80 mg | Placebo | 312 | 68-79% | 21 (20 mg), 23 (40 mg), 23 (80 mg) |
| [37] | RCT | Eletriptan 40 mg | Naratriptan 2.5 mg or placebo | 483 | 79-82% | 15 (ele), 27 (nara) |
| [40] | RCT | eletriptan 20 mg, 40 mg and 80 mg | Sumatriptan 100 mg or placebo | 692 | 80-85% | 29 |
| [97] | RCT | Sumatriptan 100 mg | Placebo | 61 | NR | 41 |
| [98] | RCT | Frovatriptan 2.5, 5, 10, 20, and 40 mg | Placebo | 635 | 508~~,~~ (73.1%) | 33.3 |
| [125] | RCT | Sumatriptan 50 mg | acetaminophen 500 mg, aspirin 500 mg, and caffeine 130 mg | 188 | 81% | 11.9 |
| [126] | RCT | Sumatriptan 6 mg sc | Placebo | 108 | M/F ratio 1:9 | 6 |
| [44] | RCT | sumatriptan 100 mg tablets | Placebo | 253 | 184 (72.7%) | 42 |
| [101] | RCT | Eletriptan 40 mg or sumatriptan 100 mg | Placebo | 2113 | 86-87% | 20 (eletriptan), 27 (sumatriptan) |
| [102] | RCT | Sumatriptan 100 mg | Placebo | 233 | 198 (85.0%) | 19 |
| [50] | RCT | Frovatriptan 2.5 mg | Placebo | 229 | 207 (90.4%) | 50 |
| [127] | RCT | Sumatriptan 6 mg sc | Placebo | 209 | 189 (90.4%) | 42 |
| [58] | RCT | Zolmitriptan 1,2.5,5 mg | Placebo | 202 | 150 (74.3%) | 5.6 to 17.3 |
| [59] | RCT | Eletriptan 40 mg / 80 mg | Sumatriptan 50 mg / 100 mg | 774 | NR | 13 to 28 |
| [103] | RCT | Rizatriptan 2.5 mg / 5 mg / 10 mg | Placebo | 249 | 222 (89%) | 8.1 to 11.8 |
| [104] | RCT | Sumatriptan 100 mg | Placebo | 1440 | 1226 (85.1%) | 13 to 22 |
| [60] | RCT | Rizatriptan 10 mg oral disintegrating tablets | Placebo | 100 | 92 (92.0%) | 50 |
| [61] | RCT | Rizatriptan 10 mg oral disintegrating tablets | Placebo | 102 | 88 (86.3%) | 39 |
| [6] | RCT | Eletriptan 20 mg, 40 mg, 80 mg oral | Placebo | 1190 | 85-90% | 29 (20-mg), 26 (40-mg), 21 (80-mg) |
| [62] | RCT | Sumatriptan Fast-Disintegrating/Rapid-Release Formulation tablets 50 mg or 100 mg | Placebo | 3331 | 84-88% | 41-43 (50 mg), 37-39 (100 mg) |
| [7] | RCT | sumatriptan 50 mg oral | naproxen sodium 500 mg, combination of sumatriptan 50 mg and naproxen sodium 500 mg, or placebo | 972 | 880 (90.5%) | 51 |
| [64] | RCT | Zolmitriptan 5 mg ODT | Placebo | 670 | 85.4-87.7% | 53.1 |
| [66] | RCT | Eletriptan 40 and 80 mg | Zolmitriptan 2.5 mg or placebo | 1312 | 83-88% | 14 (ele 80 mg), 26 (zolmi), 20 (ele 40 mg) |
| [102] | RCT | Sumatriptan 100 mg | Placebo | 232 | 83-86% | 19 |
| [8] | RCT | frovatriptan plus dexketoprofen (25 mg or 37.5 mg) | frovatriptan 2.5 mg | 279 | 248 (89%) | 45 (frova), 33 (FroDex25), 29 (FroDex37.5) |
| [73] | RCT | Sumatriptan intranasal 20 mg | Placebo | 56 | 48 (85.7%) | 1/26, 3.8 |
| [128] | RCT | Sumatriptan 100 mg RT | Placebo | 35 | 63% | 19 |
| [74] | RCT | Zolmitriptan nasal spray 0.5, 2.5, or 5 mg | Placebo | 798 | 493 (61.8%) | 20.3 |
| [76] | RCT | Eletriptan 40 mg | Placebo | 274 | 56-59% | 32 |
| [129] | Prospective cohort study | Sumatriptan 100 mg p.o. | - | 63 | 44 (70%) | Of the 10 poor responders, 50% took rescue medication. |
| [78] | Prospective, open-label, pilot cohort study | Frovatriptan 2.5 mg | - | 20 | 100% | Rescue medication: 35%, second dose of frovatriptan: 50%. |
| [81] | Long-term, open-label, single-arm, prospective study | Almotriptan 12.5 mg p.o. | - | 420 | 233 (55.5%) | 334 patients (79.5%) for 1 or more migraines during the course of the study. |
| [82] | Open-label, single-arm, prospective study | Sumatriptan 6 mg s.c. | - | PP: 212; safety: 242 | PP: 82.5%; safety: 81.0% | 15.1% of all attacks. |
| [9] | Open-label, single-arm study | Eletriptan 40 mg p.o. | - | 113 | 81% | 10 |
| [10] | Open-label, single-arm study | Eletriptan 40 mg p.o. | - | 110 | 75% | 16 |
| [24] | Cohort study | Almotriptan 12.5 mg p.o. | - | 456 | 78% | 15 (early treatment), 27 (late treatment) |
| [105] | Prospective observational study | frovatriptan 2.5 mg | - | 1620 | 1227 (80.8%) | 3.88 (early treatment), 13.73 (late treatment) |
| [84] | Open-label, single-arm study | Eletriptan 40 mg | - | 123 | 80% | 14.9 |
| [130] | open, multi-centre study | Sumatriptan 50 mg and 100 mg oral | - | 163 | 131 (80.4%) | 21.9 |
| [106] | Long-term, open-label study | Frovatriptan 2.5-7.5 mg oral | - | 486 | 438 (88.3%) | 12 |
| [87] | Real-life, open-label, non-comparative outpatient study | Eletriptan | - | 611 | 86.2% | 6,40 |
| [128] | Open-label long-term study | Sumatriptan with RT technology 100 mg | - | 32 | 63% | 19% of attacks |

**Table S5**. Response in triptan non-responders

| **Study** | **Design** | **Active drug** | **Comparator** | **Patients (n)** | **Women (n, %)** | **Previously failed triptan(s)** | **Responders with previous triptan failure, n (%)** |
| --- | --- | --- | --- | --- | --- | --- | --- |
| [13] | RCT | Eletriptan 40/80 mg p.o. | Placebo | 971 | 83% | Previous treatment with a triptan in 72.5% (failure status unknown). | In non-responders to ELE-40: 42.5-60% responder with ELE-80. |
| [117] | RCT | Rizatriptan 10 mg p.o. | Placebo | 80 | 67 (83.8%) | 61.5% were prior triptan users (failure status unknown). | - |
| [28] | RCT | Almotriptan 12.5 mg p.o. | Placebo | 198 | 85.8-88.0% | Sumatriptan 50 mg: 100% | Pain freedom at 2 hours in patients with moderate pain: 36.7%; with severe pain: 31.9%.  Sustained pain freedom at 24 hours: 20.9%.  Pain relief at 2 hours in patients with moderate pain: 50%; with severe pain: 46.4%. |
| [29] | RCT | Almotriptan 12.5 mg p.o. | Placebo | 221 | 85.8-88% | Sumatriptan 50 mg: 100% | Pain at 2 hours: 47.5%.  Pain freedom at 2 hours: 33.3%.  Sustained pain freedom at 24 hours: 20.9%.  Rescue medication: 26.6%. |
| [48] | RCT | Zolmitriptan 5mg p.o. | Rizatriptan 10mg | 48 | NR | 100% sumatriptan non-responders | Only 19% remained non-responder to both zolmitriptan and rizatriptan |
| [131] | RCT | Sumatriptan 50mg + metoclopramide 10mg p.o. | Sumatriptan 50mg | 16 | 13 (81.3) | 100% triptan non-responders | Headache response: 44% (combination) vs 31% (sumatriptan alone) |
| [61] | RCT | Rizatriptan 10mg ODT | Placebo | 102 | 88 (86.3) | 100% sumatriptan non-responders | Pain relief at 2h: 51% Pain-free at 2h: 22% Sustained pain relief at 24h: 38% Sustained pain freedom at 24h: 20%. rescue medication: 39%. |
| [65] | RCT | Naratriptan 2.5 mg p.o. | Sumatriptan 50 mg oral or placebo | 347 | 90% | Sumatriptan oral (63.4%) | 63.4% were poor responders to sumatriptan at 4h. In these poor responders, headache relief at 4h was present in 41% and no pain at 4h was observed in 22%. |
| [132] | Postmarketing survey study | Frovatriptan 2.5 mg p.o. | - | 5025 | 82% | 28.2% with previous triptan use; 14.3% with previous triptan+NSAID use. | 44.2% "very good or good" effectiveness of frovatriptan after "poor or satisfactory" response to other triptans. 61.0% "very good or good" effectiveness of frovatriptan after "poor or satisfactory" response to other triptans+NSAIDs. |
| [9] | Open-label, single-arm study | Eletriptan 40 mg p.o. | - | 113 | 81% | - | At 24 hours postdose, 74% of patients rated eletriptan as preferable to NSAIDs (and/or any previous treatment for migraine). Inclusion criterium was use of NSAIDs to treat previous migraines. |
| [83] | Open-label, single-arm study | Sumatriptan 6 mg s.c. | - | 43 | - | 100% | Pain relief at 2 hours: 91%. Pain freedom at 2 hours: 56%. 24-h sustained pain response: 32%. |
| [84] | Open-label, single-arm study | Eletriptan 40 mg p.o. | - | 123 | 80% | Rizatriptan (n=103) | Among the patients with inadequate headache response to rizatriptan (n=103), 61.5% reported a headache response to eletriptan at 2h. Among the subgroup of patients (n = 56) who cited recurrence as one of the reasons for dissatisfaction with rizatriptan therapy, 58.9% reported no headache recurrence at 24h. |

**Table S6**. Comparisons of different triptan formulations.

| **Study** | **Design** | **Non-oral formulation** | **Oral formulation** | **No. of patients (non-oral)** | **No. of patients (oral)** | **Pain relief at 2 hours, n (%), non-oral** | **Pain relief at 2 hours, n (%), oral** |
| --- | --- | --- | --- | --- | --- | --- | --- |
| [133] | RCT | Zolmitriptan 0.5 / 1 / 2.5 / 5 mg intranasal | Zolmitriptan 2.5 mg p.o. | 916 | 230 | Reduced intensity of migraine pain (using a scale of none, mild, moderate or severe) from severe or moderate at baseline to mild or no pain at 2 hours after treatment: 41.5% (0.5 mg), 54.8% (1 mg), 58.6% (2.5 mg), 70.3% (5 mg) vs. 61.3% (2.5 mg p.o.) – significant higher response with 5 mg nasal vs. 2.5 mg p.o. | |
| [134] | RCT | Sumatriptan 6 mg s.c. | Sumatriptan 100 mg p.o. | 149 | 156 | Headache relief at 2h for attack 1: 66-76% with oral vs. 72-87% with sc.  >70% of patients experience headache relief with both oral and sc at 4h. 97% of patients had relief with at least one of the formulations at 4h. Of patients who responded to one formulation only, a higher proportion responsed to sc than oral. Response to sc was significantly superior to oral at 4h for those who had relief from only 1 formulation. | |
| [135] | RCT | Sumatriptan 22 mg intranasal | Sumatriptan 100 mg p.o. | 131 | 128 | Attacks treated with AVP-825 showed significantly lower mean pain intensity and mean disability from 10 to 90 minutes post-dose (effect sizes ranged from −0.09 to −0.29 and P values ranged from P < .0001 to P = .01). Within-person variability (i.e. consistency) was significantly greater at 10–15 minutes but significantly reduced from 45 to 120 minutes for attacks treated with AVP-825 compared to oral sumatriptan, indicating more rapid onset of action. | |
| [136] | RCT | Sumatriptan 22 mg intranasal | Sumatriptan 100 mg p.o. | 138 | 137 | Migraine freedom at 2 hours: 60.7%. | Migraine freedom at 2 hours: 56.7%. |
| [68] | RCT | Sumatriptan 22 mg intranasal | Sumatriptan 100 mg p.o. | 185 | 185 | Pain relief at 2 hours: 79.6%. | Pain relief at 2 hours: 72.0% (non significant). |
| [137] | Open-label, real-world | Rizatriptan 10 mg ODT | Rizatriptan 10 mg p.o. | 3532 | 3565 | Pain-free 2h postdose: 31.3%. Pain-free or mild pain 2h postdose: 66.5%. | Pain-free 2h postdose: 30.8%. Pain-free or mild pain 2h postdose: 65.9% tablet. |
| [138] | Naturalistic study | Rizatriptan 10 mg ODT | Rizatriptan 10 mg p.o. | 216 | 216 | Pain free or only mild pain at 2 hours: 63.9%. | Pain free or only mild pain at 2 hours: 61.6%. |
| [139] | Survey | Sumatriptan 6 mg s.c. | Sumatriptan 100 mg p.o. | 281 | 198 | Headache relief in at least 4/5 attacks: 69%. | Headache relief in at least 4/5 attacks: 52%. |

**Table S7**. Comparisons between early and late treatment with triptans

| **Study** | **Design** | **Drug(s)** | **Definition of early treatment** | **Definition of late treatment** | **No. of patients** | **Pain relief at 2 hours, n (%), early treatment** | **Pain relief at 2 hours, n (%), late treatment** |
| --- | --- | --- | --- | --- | --- | --- | --- |
| [140] | RCT | Sumatriptan 6/8 mg s.c. | Duration of attack before treatment ≤ 240 minutes | Duration of attack before treatment >240 minutes | 639 | Similar response treated early and late. After 60 minutes: 75% (6 mg), 73% (8 mg) | After 60 minutes: 71% (6 mg), 81% (8 mg). |
| [11] | RCT | Sumatriptan 100/200/300 mg ODT | Duration of attack before treatment ≤ 240 minutes | Duration of attack before treatment >240 minutes | 1092 | Similar response rates when treated early and late. | |
| [141] | RCT | Sumatriptan 6 mg s.c. | Duration of attack before treatment ≤ 240 minutes | Duration of attack before treatment >240 minutes | 235 | Similar response rates when treated early and late. | |
| [11] | RCT | Sumatriptan 100 mg ODT | Duration of attack before treatment ≤ 240 minutes | Duration of attack before treatment >240 minutes | 233 | 50% | 53% |
| [118] | RCT | Frovatriptan 2.5 mg p.o. | At the onset on mild migraine pain | At least 2 h later if the headache progressed to  moderate/severe. | 275 | Pain freedom at 2 hours with early use: 28%. Significantly fewer patients required a second dose of study medication or rescue medication in the 24 h following early use of frovatriptan (50%) than those who used frovatriptan later (68% p < 0.001). In the early use group, 80% of patients did not use rescue medication in the 24 h after dosing. The 24-h sustained pain free response, defined as pain free with no re-medication and no recurrence, was significantly higher following early use of frovatriptan compared to later use, 40% vs 31% (p < 0.05). Frovatriptan early use provided a longer pain free response, with a median duration of 19.8 h compared to 17.6 h following later use. | |
| [142] | RCT | Sumatriptan 50 mg p.o. | Treating while pain was mild | Treating while pain was already moderate to severe | 26 | Pain freedom at 2 hours in 50% of all attacks | Pain freedom at 2 hours in 27% of all attacks |
| [143] | RCT | Sumatriptan 50/100 mg p.o. | Treating while pain was mild | Treating while pain was already moderate to severe | 92 | Pain freedom at 2 hours: 51% (50 mg), 67% (100 mg). | Pain freedom at 2 hours: 31% (50 mg), 36% (100 mg). |
| [24] | RCT | Almotriptan 12.5 mg p.o. | Treating while pain was mild | Treating while pain was already moderate to severe | 404 | Pain freedom at 2 hours in patients with early/mild migraine with allodynia: 53.9% and without allodynia: 52.5%. | Pain freedom at 2 hours in patients with moderate/severe migraine with allodynia: 31.4% and without allodynia: 44.3%. |
| [144] | RCT | Almotriptan 12.5 mg p.o. | Treating at earliest onset of headache pain, within 1 hour | Treating when headache pain  intensity is moderate or severe | 1304 | Total headache duration: 3.18h. 2h pain free: 42.5%. Sustained pain free: 17.3%. Use of rescue medication: 35.5%. | Total headache duration: 5.53h ST. 2h pain free: 39.0%. Sustained pain free: 15.3%. Use of rescue medication: 36.9%. |
| [96] | RCT | Almotriptan 12.5 mg p.o. | Treatment administration when pain intensity  was mild and within 1 h of headache onset | Treating when headache pain  intensity is moderate or severe | 403 | Pain-free at 2h: 53.5%. Sustained pain-free at 24h: 46%. Headache recurrence at 24h: 6%. Median duration of pain: 2h. | Pain-free at 2h: 37.5%. Sustained pain-free at 24h: 30%. Headache recurrence at 24h: 24%. Median duration of pain: 5h. |
| [145] | RCT | Almotriptan 12.5 mg p.o. | Treatment administration when pain intensity  was mild and within 1 h of headache onset | Treating when headache pain  intensity is moderate or severe | 491 | Pain free at 2 h: 49%.  Sustained pain-free: 45.6% | Pain free at 2 h: 40%.  Sustained pain-free: 30.5%. |
| [146] | RCT | Sumatriptan 1/2/3 mg s.c. | Duration of attack before treatment ≤ 240 minutes | Duration of attack before treatment >240 minutes | 685 | 38% for sumatriptan 1 mg, 49% for 2 mg and 66% for sumatriptan 3 mg. | 39% for sumatriptan 1 mg, 42% for 2 mg and for sumatriptan 3 mg. |
| [24] | Cohort study | Almotriptan 12.5 mg p.o. | Treatment within 1 hour of onset | Treatment after 1 hour of onset | 456 | Pain freedom at 2 hours: 62%. Sustained pain-free at 24 hours: 59%. Rescue medication: 15%. | Pain freedom at 2 hours in later attacks: 35%. Sustained pain-free at 24 hours in late attacks: 33%. Rescue medication: 27%. |
| [147] | Real-world study | Rizatriptan 10 mg p.o. | Treating immediately after experiencing headache. | Treating after headache  evolved to become moderate or severe. | 1919 | Onset of headache relief within 30 min: 23%. Being largely symptom-free within 1 h: 26%. Returning to usual activities within 1h: 28%. No pain or mild pain at 2h: 65%. | Onset of headache relief within 30 min: 18%. Being largely symptom-free within 1 h: 22%. Returning to usual activities within 1h: 23%. No pain or mild pain at 2h: 67%. |
| [148] | Cross-sectional survey | Any triptan | Self-reported | Self-reported | 502 | Percent of migraine attacks requiring a second non-triptan medication: 24.9%.  Percent of migraine attacks requiring a second triptan medication: 27.2%. | Percent of migraine attacks requiring a second non-triptan medication: 36.0%.  Percent of migraine attacks requiring a second triptan medication: 25.3%. |
| [149] | Prospective | Almotriptan 12.5 mg p.o. | Treatment administration when pain intensity  was mild and within 1 h of headache onset | Treating when headache pain  intensity is moderate or severe | 436 | 2h pain-free: 62%.  Sustained pain-free: 55%. | 2h pain-free: 37%.  Sustained pain-free: 32%. |
| [150] | Prospective open-label | Sumatriptan 6 mg s.c. | As early as possible | As late as the patients could bear | 20 | Pain-free at 1h: 42%.  Pain-free at 2h: 62%. | Pain-free at 1h: 28%.  Pain-free at 2h: 55%. |

**Table S8**. Agreement on questions submitted in Round 1.

| **Question** | **N (%)** | **Decision** |
| --- | --- | --- |
| *1. Which is the most important time point to assess response to triptans?* |  | The time point mostly assessed by literature is 2 hours; a 1-hour time point is recognized as the most clinically significant by most Authors. |
| 1 hour or less | 5 (33.3) |  |
| 2 hours | 10 (66.7) |  |
| 4 hours or more | - |  |
| *2. Is pain relief (headache intensity decrease from moderate/severe to mild) acceptable to consider a triptan as effective or should pain-free status be achieved?* |  | Pain freedom should best be achieved; pain relief could be acceptable for some patients. |
| Pain relief is an acceptable goal | 7 (46.7) |  |
| Pain freedom should be achieved | 8 (53.3) |  |
| *3. Is headache recurrence within 24 hours a valid marker of triptan non-response?* |  | Given the lack of consensus, headache recurrence within 24 hours is not necessary for the definition of non-response to triptans. |
| Yes | 6 (40.0) |  |
| No | 9 (60.0) |  |
| *4. Is the use of rescue medication within 24 hours a valid marker of triptan non-response?* |  | Given the lack of consensus, use of rescue medication within 24 hours is not necessary for the definition of non-response to triptans. |
| Yes | 9 (60.0) |  |
| No | 6 (40.0) |  |
| *5. How many attacks should be treated with the same triptan before declaring its failure?* |  | Three migraine attacks should be treated before declaring non-response to a triptan. |
| 1 | - |  |
| 2 | 2 (13.3) |  |
| 3 | 12 (80.0) |  |
| More than 3 | 1 (6.7) |  |
| *6. Is triptan failure only lack of efficacy or also lack of tolerability?* |  | Triptan non-response can be declared when either efficacy or tolerability are lacking. |
| Only lack of efficacy | 3 (20.0) |  |
| Lack of efficacy or lack of tolerability | 12 (80.0) |  |
| *7. Is it justified to try a second triptan if the first one fails?* |  | A second triptan should be tried if the first one fails. |
| Yes | 15 (100.0) |  |
| No | - |  |
| *8. Is it justified to try a third triptan if two failed?* |  | A third triptan should be tried if the second one fails. |
| Yes | 12 (80.0) |  |
| No | 3 (20.0) |  |
| *9. Is it justified to try more than three triptans in one patients?* |  | Trial of three triptans is sufficient to declare non-response to triptans. |
| Yes | 4 (26.7) |  |
| No | 11 (73.3) |  |
| *10. To be defined as triptan-resistant, should failure to non-oral formulation be mandatory?* |  | Given the lack of consensus, non-response to oral formulation could be considered enough to declare triptan non-response. |
| Yes | 7 (46.7) |  |
| No | 8 (53.3) |  |
| *11. To be defined as triptan-resistant, should failure to s.c. sumatriptan be mandatory?* |  | Given the lack of consensus, non-response to s.c. sumatriptan could be considered as not necessary to declare triptan non-response. |
| Yes | 6 (40.0) |  |
| No | 9 (60.0) |  |
| *12. Should patients be assessed for early treatment with triptans before declaring the failure of a triptan?* |  | Before declaring non-response to a triptan, physicians should ensure that patients take that triptan as early as possible. |
| Yes | 14 (93.3) |  |
| No | 1 (6.7) |  |
| *13. Which is the preferable terminology among those reported below?* |  | The term “non-responders” could be preferable. |
| Triptan non-responder | 9 (60.0) |  |
| Triptan resistant | 5 (33.3) |  |
| Triptan refractory | 1 (6.7) |  |

**Table S9**. Conflicts of interest of the authors

| Name | **Employment in Pharma industry** | **Personal fees (fees as advisor or speaker, consultancy, any other)** | **Institutional fees (research support, any other)** | **Stock Option in Pharma Industry** | **Intellectual (participation in study design or analyses of one of the RCTs included in this guideline)** |
| --- | --- | --- | --- | --- | --- |
| Simona Sacco | No | Allergan-AbbVie, Abbott, AstraZeneca, Lilly, Lundbeck, Novartis, Novo-Nordisk, Teva | Novartis, Uriach | No | No |
| Christian Lampl | No | Allergan-Abbvie, Lilly, Lundbeck, Merck, Novartis, Pfizer, TEVA | No | No | No |
| Faisal Mohammad Amin | No | Lundbeck, Novartis, Teva and Eli Lilly | No | No | No |
| Mark Braschinsky | No | No | No | No | No |
| Christina Deligianni | No | No | No | No | No |
| Derya Uludüz | No | Allergan-AbbVie, Eli Lilly, Novartis | No | No | Investigator in trials by Amgen, Novartis |
| Jan Versijpt | No | Allergan-AbbVie, Lundbeck, Novartis, Teva | No | No | No |
| Anne Ducros | No | Allergan-Abbvie, Novartis, Lundbeck, Lilly,Pfizer TEVA, | Pfizer | no | no |
| Raquel Gil-Gouveia | No | Allergan-Abbvie, Lilly, Lunbeck, Novartis, Teva | Novartis, Amgen, Lundbeck, Bayer, | No | No |
| Zaza Katsarava | No | Allergan-Abbvie, Lilly, Lundbeck, Merck, Novartis, TEVA | Novartis | No | No |
| Paolo Martelletti | No | Springer, SpringerNature | Novartis | No | No |
| Raffaele Ornello | No | Eli Lilly, Novartis, Teva | Allergan-AbbVie, Novartis | No | No |
| Bianca Raffaelli | No | Allergan-Abbvie, Hormosan, Lilly, Novartis, TEVA | Novartis | No | No |
| Deirdre Boucherie | No | No | No | No | No |
| Patricia Pozo-Rosich | No | AbbVie, Biohaven, Eli Lilly, Lundbeck, Novartis, Pfizer, Teva | AbbVie, Novartis, Teva | No | No |
| Margarita Sanchez del Rio | No | Allergan-AbbVie, Lilly, Novartis, TEVA | Lundbeck, Novartis, Teva and Eli Lilly | No | No |
| Alexandra J Sinclair | Director and Chief Scientific officer for Invex Therapeutics (a University of Birmingham start-up company running a trial in Idiopathic Intracranial Hypertension) | Allergan-AbbVie, Amgen, Chiesi, Novartis, Lundbeck | No | Invex Therapeutics (a University of Birmingham start-up company running a trial in Idiopathic Intracranial Hypertension) | No |
| Antoinette Maassen van den Brink | No | Allergan-Abbvie, Lilly, Novartis, Teva | Novartis, Satsuma | No | No |
| Uwe Reuter | No | Amgen, Allergan-AbbVie, Alder, Lilly, Lundbeck, Novartis, Pfizer, Teva | BMBF, Novartis Pharma (Cherub 01); Amgen, Allergan-AbbVie, Alder, Lilly, Lundbeck, Pfizer, Teva | No | Principal investigator for trials sponsired by Eli Lilly, Lundbeck, Novartis, Pfizer |

**References**

1. Dodick, D., et al., *Speed of onset, efficacy and tolerability of zolmitriptan nasal spray in the acute treatment of migraine: a randomised, double-blind, placebo-controlled study.* CNS Drugs, 2005. **19**(2): p. 125-36.

2. Dowson, A.J., et al., *Zolmitriptan orally disintegrating tablet is effective in the acute treatment of migraine.* Cephalalgia, 2002. **22**(2): p. 101-6.

3. Gallagher, R.M., et al., *A comparative trial of zolmitriptan and sumatriptan for the acute oral treatment of migraine.* Headache, 2000. **40**(2): p. 119-28.

4. Rapoport, A.M., et al., *Optimizing the dose of zolmitriptan (Zomig, 311C90) for the acute treatment of migraine. A multicenter, double-blind, placebo-controlled, dose range-finding study. The 017 Clinical Trial Study Group.* Neurology, 1997. **49**(5): p. 1210-8.

5. Ryan, R.E.J., et al., *Efficacy of Zolmitriptan at Early Time-Points for the Acute Treatment of Migraine and Treatment of Recurrence.* CNS Drugs, 2000. **13**(3): p. 215-226.

6. Sheftell, F., et al., *Efficacy, safety, and tolerability of oral eletriptan for treatment of acute migraine: a multicenter, double-blind, placebo-controlled study conducted in the United States.* Headache, 2003. **43**(3): p. 202-13.

7. Smith, T.R., et al., *Sumatriptan and naproxen sodium for the acute treatment of migraine.* Headache, 2005. **45**(8): p. 983-91.

8. Tuchman, M., et al., *Efficacy and tolerability of zolmitriptan oral tablet in the acute treatment of menstrual migraine.* CNS Drugs, 2006. **20**(12): p. 1019-26.

9. Chia, Y.C., et al., *Efficacy of eletriptan in migraineurs with persistent poor response to nonsteroidal anti-inflammatory drugs.* Headache, 2003. **43**(9): p. 984-90.

10. Diamond, M.L., et al., *Effectiveness of eletriptan in acute migraine: primary care for Excedrin nonresponders.* Headache, 2004. **44**(3): p. 209-16.

11. *Evaluation of a multiple-dose regimen of oral sumatriptan for the acute treatment of migraine. The Oral Sumatriptan International Multiple-Dose Study Group.* Eur Neurol, 1991. **31**(5): p. 306-13.

12. Eletriptan Steering Committee in, J., *Efficacy and safety of eletriptan 20 mg, 40 mg and 80 mg in Japanese migraineurs.* Cephalalgia, 2002. **22**(6): p. 416-23.

13. Almas, M., et al., *Consistency of eletriptan in treating migraine: Results of a randomized, within-patient multiple-dose study.* Cephalalgia, 2014. **34**(2): p. 126-35.

14. Asadollahi, S., et al., *Promethazine plus sumatriptan in the treatment of migraine: a randomized clinical trial.* Headache, 2014. **54**(1): p. 94-108.

15. Bigal, M., et al., *A randomized double-blind study comparing rizatriptan, dexamethasone, and the combination of both in the acute treatment of menstrually related migraine.* Headache, 2008. **48**(9): p. 1286-93.

16. Bigal, M.E., et al., *Sumatriptan Iontophoretic Transdermal System Reduces Treatment-Emergent Nausea and Is Effective in Patients With and Without Nausea at Baseline - Results From a Randomized Controlled Trial.* Headache, 2015. **55**(8): p. 1124-32.

17. Brandes, J.L., et al., *Sumatriptan-naproxen for acute treatment of migraine: a randomized trial.* JAMA, 2007. **297**(13): p. 1443-54.

18. Burke-Ramirez, P., et al., *Efficacy and tolerability of subcutaneous sumatriptan for acute migraine: a comparison between ethnic groups.* Headache, 2001. **41**(9): p. 873-82.

19. Cabarrocas, X. and G. Almotriptan Study, *Efficacy and tolerability of subcutaneous almotriptan for the treatment of acute migraine: a randomized, double-blind, parallel-group, dose-finding study.* Clin Ther, 2001. **23**(11): p. 1867-75.

20. Christoph-Diener, H., et al., *Predicting the response to sumatriptan: the Sumatriptan Naratriptan Aggregate Patient Database.* Neurology, 2004. **63**(3): p. 520-4.

21. Dahlof, C., et al., *Zolmitriptan, a 5-HT1B/1D receptor agonist for the acute oral treatment of migraine: a multicentre, dose-range finding study.* Eur J Neurol, 1998. **5**(6): p. 535-543.

22. Dahlof, C., et al., *Dose finding, placebo-controlled study of oral almotriptan in the acute treatment of migraine.* Neurology, 2001. **57**(10): p. 1811-7.

23. Dahlof, C.G., et al., *Within-patient consistency of response of rizatriptan for treating migraine.* Neurology, 2000. **55**(10): p. 1511-6.

24. Diaz-Insa, S., et al., *The impact of allodynia on the efficacy of almotriptan when given early in migraine: data from the "Act when mild" study.* Int J Neurosci, 2011. **121**(12): p. 655-61.

25. Di Monda, V., et al., *Efficacy of a fixed combination of indomethacin, prochlorperazine, and caffeine versus sumatriptan in acute treatment of multiple migraine attacks: a multicenter, randomized, crossover trial.* Headache, 2003. **43**(8): p. 835-44.

26. Diamond, M.L., et al., *Characteristics of migraine attacks and responses to almotriptan treatment: a comparison of menstrually related and nonmenstrually related migraines.* Headache, 2008. **48**(2): p. 248-58.

27. Diener, H.C., *Efficacy and safety of intravenous acetylsalicylic acid lysinate compared to subcutaneous sumatriptan and parenteral placebo in the acute treatment of migraine. A double-blind, double-dummy, randomized, multicenter, parallel group study. The ASASUMAMIG Study Group.* Cephalalgia, 1999. **19**(6): p. 581-8; discussion 542.

28. Diener, H.C., *Efficacy of almotriptan 12.5 mg in achieving migraine-related composite endpoints: a double-blind, randomized, placebo-controlled study in patients controlled study in patients with previous poor response to sumatriptan 50 mg.* Curr Med Res Opin, 2005. **21**(10): p. 1603-10.

29. Diener, H.C., et al., *Almotriptan in migraine patients who respond poorly to oral sumatriptan: a double-blind, randomized trial.* Headache, 2005. **45**(7): p. 874-82.

30. Diener, H.C., et al., *Efficacy, tolerability and safety of oral eletriptan and ergotamine plus caffeine (Cafergot) in the acute treatment of migraine: a multicentre, randomised, double-blind, placebo-controlled comparison.* Eur Neurol, 2002. **47**(2): p. 99-107.

31. Diener, H.C., et al., *The efficacy and safety of sc alniditan vs. sc sumatriptan in the acute treatment of migraine: a randomized, double-blind, placebo-controlled trial.* Cephalalgia, 2001. **21**(6): p. 672-9.

32. Dowson, A.J., et al., *Tolerability and consistency of effect of zolmitriptan nasal spray in a long-term migraine treatment trial.* CNS Drugs, 2003. **17**(11): p. 839-51.

33. Dowson, A.J., et al., *Almotriptan is an effective and well-tolerated treatment for migraine pain: results of a randomized, double-blind, placebo-controlled clinical trial.* Cephalalgia, 2002. **22**(6): p. 453-61.

34. Facchinetti, F., et al., *The efficacy and safety of subcutaneous sumatriptan in the acute treatment of menstrual migraine. The Sumatriptan Menstrual Migraine Study Group.* Obstet Gynecol, 1995. **86**(6): p. 911-6.

35. Farkkila, M., et al., *Eletriptan for the treatment of migraine in patients with previous poor response or tolerance to oral sumatriptan.* Cephalalgia, 2003. **23**(6): p. 463-71.

36. Franconi, F., et al., *Gender and triptan efficacy: a pooled analysis of three double-blind, randomized, crossover, multicenter, Italian studies comparing frovatriptan vs. other triptans.* Neurol Sci, 2014. **35 Suppl 1**: p. 99-105.

37. Garcia-Ramos, G., et al., *Comparative efficacy of eletriptan vs. naratriptan in the acute treatment of migraine.* Cephalalgia, 2003. **23**(9): p. 869-76.

38. Gawel, M., et al., *Zolmitriptan 5 mg nasal spray: efficacy and onset of action in the acute treatment of migraine--results from phase 1 of the REALIZE Study.* Headache, 2005. **45**(1): p. 7-16.

39. Geraud, G., et al., *Zolmitriptan versus a combination of acetylsalicylic acid and metoclopramide in the acute oral treatment of migraine: a double-blind, randomised, three-attack study.* Eur Neurol, 2002. **47**(2): p. 88-98.

40. Goadsby, P.J., et al., *Eletriptan in acute migraine: a double-blind, placebo-controlled comparison to sumatriptan. Eletriptan Steering Committee.* Neurology, 2000. **54**(1): p. 156-63.

41. Goldstein, J., et al., *Crossover comparison of rizatriptan 5 mg and 10 mg versus sumatriptan 25 mg and 50 mg in migraine. Rizatriptan Protocol 046 Study Group.* Headache, 1998. **38**(10): p. 737-47.

42. Hamalainen, M.L., K. Hoppu, and P. Santavuori, *Sumatriptan for migraine attacks in children: a randomized placebo-controlled study. Do children with migraine respond to oral sumatriptan differently from adults?* Neurology, 1997. **48**(4): p. 1100-3.

43. Ho, T.W., et al., *Efficacy and tolerability of rizatriptan in pediatric migraineurs: results from a randomized, double-blind, placebo-controlled trial using a novel adaptive enrichment design.* Cephalalgia, 2012. **32**(10): p. 750-65.

44. Kaniecki, R., et al., *Prevalence of migraine and response to sumatriptan in patients self-reporting tension/stress headache.* Curr Med Res Opin, 2006. **22**(8): p. 1535-44.

45. Lewis, D.W., et al., *Efficacy of zolmitriptan nasal spray in adolescent migraine.* Pediatrics, 2007. **120**(2): p. 390-6.

46. Lipton, R.B., et al., *2000 Wolfe Award. Sumatriptan for the range of headaches in migraine sufferers: results of the Spectrum Study.* Headache, 2000. **40**(10): p. 783-91.

47. Loder, E., et al., *Preference comparison of rizatriptan ODT 10-mg and sumatriptan 50-mg tablet in migraine.* Headache, 2001. **41**(8): p. 745-53.

48. Mathew, N.T., et al., *Treatment of nonresponders to oral sumatriptan with zolmitriptan and rizatriptan: a comparative open trial.* Headache, 2000. **40**(6): p. 464-5.

49. Mauskop, A., et al., *Zolmitriptan is effective for the treatment of persistent and recurrent migraine headache.* Curr Med Res Opin, 1999. **15**(4): p. 282-9.

50. Moon, H.S., et al., *Frovatriptan is Effective and Well Tolerated in Korean Migraineurs: A Double-Blind, Randomized, Placebo-Controlled Trial.* J Clin Neurol, 2010. **6**(1): p. 27-32.

51. Pascual, J., et al., *Consistent efficacy and tolerability of almotriptan in the acute treatment of multiple migraine attacks: results of a large, randomized, double-blind, placebo-controlled study.* Cephalalgia, 2000. **20**(6): p. 588-96.

52. Peikert, A., et al., *Sumatriptan nasal spray: a dose-ranging study in the acute treatment of migraine.* Eur J Neurol, 1999. **6**(1): p. 43-9.

53. Rao, A.S., et al., *A Randomized Trial of Ketorolac vs. Sumatripan vs. Placebo Nasal Spray (KSPN) for Acute Migraine.* Headache, 2016. **56**(2): p. 331-40.

54. Rapoport, A., et al., *Dose range-finding studies with frovatriptan in the acute treatment of migraine.* Headache, 2002. **42 Suppl 2**: p. S74-83.

55. Rothner, A.D., et al., *Zolmitriptan oral tablet in migraine treatment: high placebo responses in adolescents.* Headache, 2006. **46**(1): p. 101-9.

56. Ryan, R., et al., *Clinical efficacy of frovatriptan: placebo-controlled studies.* Headache, 2002. **42 Suppl 2**: p. S84-92.

57. Sakai, F., et al., *Eletriptan for the acute treatment of migraine: results of bridging a Japanese study to Western clinical trials.* Curr Med Res Opin, 2004. **20**(3): p. 269-77.

58. Sakai, F., et al., *Zolmitriptan is effective and well tolerated in Japanese patients with migraine: a dose-response study.* Cephalalgia, 2002. **22**(5): p. 376-83.

59. Sandrini, G., et al., *Eletriptan vs sumatriptan: a double-blind, placebo-controlled, multiple migraine attack study.* Neurology, 2002. **59**(8): p. 1210-7.

60. Seeburger, J.L., et al., *Rizatriptan for treatment of acute migraine in patients taking topiramate for migraine prophylaxis.* Headache, 2012. **52**(1): p. 57-67.

61. Seeburger, J.L., et al., *Efficacy and tolerability of rizatriptan for the treatment of acute migraine in sumatriptan non-responders.* Cephalalgia, 2011. **31**(7): p. 786-96.

62. Sheftell, F.D., et al., *Two replicate randomized, double-blind, placebo-controlled trials of the time to onset of pain relief in the acute treatment of migraine with a fast-disintegrating/rapid-release formulation of sumatriptan tablets.* Clin Ther, 2005. **27**(4): p. 407-17.

63. Solomon, G.D., et al., *Clinical efficacy and tolerability of 2.5 mg zolmitriptan for the acute treatment of migraine. The 042 Clinical Trial Study Group.* Neurology, 1997. **49**(5): p. 1219-25.

64. Spierings, E.L., et al., *Acute treatment of migraine with zolmitriptan 5 mg orally disintegrating tablet.* CNS Drugs, 2004. **18**(15): p. 1133-41.

65. Stark, S., et al., *Naratriptan efficacy in migraineurs who respond poorly to oral sumatriptan.* Headache, 2000. **40**(7): p. 513-20.

66. Steiner, T.J., et al., *Comparative efficacy of eletriptan and zolmitriptan in the acute treatment of migraine.* Cephalalgia, 2003. **23**(10): p. 942-52.

67. Sunshine, A., et al., *Comparative sensitivity of stopwatch methodology and conventional pain assessment measures for detecting early response to triptans in migraine: results of a randomized, open-label pilot study.* Clin Ther, 2006. **28**(8): p. 1107-1115.

68. Tepper, S.J., et al., *AVP-825 breath-powered intranasal delivery system containing 22 mg sumatriptan powder vs 100 mg oral sumatriptan in the acute treatment of migraines (The COMPASS study): a comparative randomized clinical trial across multiple attacks.* Headache, 2015. **55**(5): p. 621-35.

69. Tepper, S.J., et al., *A long-term study to maximise migraine relief with zolmitriptan.* Curr Med Res Opin, 1999. **15**(4): p. 254-71.

70. Tfelt-Hansen, P., et al., *Oral rizatriptan versus oral sumatriptan: a direct comparative study in the acute treatment of migraine. Rizatriptan 030 Study Group.* Headache, 1998. **38**(10): p. 748-55.

71. Tullo, V., et al., *Comparison of frovatriptan plus dexketoprofen (25 mg or 37.5 mg) with frovatriptan alone in the treatment of migraine attacks with or without aura: a randomized study.* Cephalalgia, 2014. **34**(6): p. 434-45.

72. Vollono, C., et al., *Multiple attack study on the available triptans in Italy versus placebo.* Eur J Neurol, 2005. **12**(7): p. 557-63.

73. Wang, S.J., J.L. Fuh, and Z.A. Wu, *Intranasal sumatriptan study with high placebo response in Taiwanese patients with migraine.* J Chin Med Assoc, 2007. **70**(2): p. 39-46.

74. Winner, P., et al., *Efficacy and tolerability of zolmitriptan nasal spray for the treatment of acute migraine in adolescents: Results of a randomized, double-blind, multi-center, parallel-group study (TEENZ).* Headache, 2016. **56**(7): p. 1107-19.

75. Winner, P., et al., *Rizatriptan 5 mg for the acute treatment of migraine in adolescents: a randomized, double-blind, placebo-controlled study.* Headache, 2002. **42**(1): p. 49-55.

76. Winner, P., et al., *Eletriptan for the acute treatment of migraine in adolescents: results of a double-blind, placebo-controlled trial.* Headache, 2007. **47**(4): p. 511-8.

77. *The long-term tolerability and efficacy of oral zolmitriptan (Zomig, 311C90) in the acute treatment of migraine. An international study. The International 311C90 Long-term Study Group.* Headache, 1998. **38**(3): p. 173-83.

78. Allais, G., et al., *Oral contraceptive-induced menstrual migraine. Clinical aspects and response to frovatriptan.* Neurol Sci, 2008. **29 Suppl 1**: p. S186-90.

79. Barbanti, P., et al., *Sumatriptan in migraine with unilateral cranial autonomic symptoms: an open study.* Headache, 2003. **43**(4): p. 400-3.

80. Becker, W.J., et al., *Topiramate prophylaxis and response to triptan treatment for acute migraine.* Headache, 2006. **46**(9): p. 1424-30.

81. Berenson, F., et al., *Long-term, open-label safety study of oral almotriptan 12.5 mg for the acute treatment of migraine in adolescents.* Headache, 2010. **50**(5): p. 795-807.

82. Cady, R.K., et al., *Satisfaction with and confidence in needle-free subcutaneous sumatriptan in patients currently treated with triptans.* Headache, 2011. **51**(8): p. 1202-11.

83. Diamond, S., et al., *Sumatriptan 6 mg subcutaneous as an effective migraine treatment in patients with cutaneous allodynia who historically fail to respond to oral triptans.* J Headache Pain, 2007. **8**(1): p. 13-8.

84. Goldstein, J., et al., *Eletriptan in migraine patients reporting unsatisfactory response to rizatriptan.* Headache, 2006. **46**(7): p. 1142-50.

85. Sheftell, F., et al., *Quantifying the return of headache in triptan-treated migraineurs: an observational study.* Cephalalgia, 2010. **30**(7): p. 838-46.

86. Tuchman, M., et al., *Zolmitriptan provides consistent migraine relief when used in the long-term.* Curr Med Res Opin, 1999. **15**(4): p. 272-81.

87. Turcani, P., et al., *Real-life experiences in migraine therapy.* Bratisl Lek Listy, 2010. **111**(2): p. 74-8.

88. Landy, S., et al., *Efficacy and safety of DFN-11 (sumatriptan injection, 3 mg) in adults with episodic migraine: an 8-week open-label extension study.* J Headache Pain, 2018. **19**(1): p. 70.

89. Landy, S.H., et al., *An open-label trial of a sumatriptan auto-injector for migraine in patients currently treated with subcutaneous sumatriptan.* Headache, 2013. **53**(1): p. 118-125.

90. Martin, V.T., et al., *Eletriptan treatment of migraine in patients switching from barbiturate-containing analgesics: results from a multiple-attack study.* Cephalalgia, 2005. **25**(9): p. 726-34.

91. Nett, R.B., et al., *Patient satisfaction with eletriptan in the acute treatment of migraine in primary care.* Int J Clin Pract, 2007. **61**(10): p. 1677-85.

92. Rothner, A.D., et al., *One-year tolerability and efficacy of sumatriptan nasal spray in adolescents with migraine: results of a multicenter, open-label study.* Clin Ther, 2000. **22**(12): p. 1533-46.

93. Rothrock, J.F., et al., *Needle-free subcutaneous sumatriptan for triptan users requiring a change in migraine therapy: efficacy and impact on patient-rated functionality, satisfaction, and confidence.* Curr Med Res Opin, 2011. **27**(11): p. 2185-91.

94. Freitag, F.G., et al., *Comparative study of a combination of isometheptene mucate, dichloralphenazone with acetaminophen and sumatriptan succinate in the treatment of migraine.* Headache, 2001. **41**(4): p. 391-8.

95. Geraud, G., et al., *Comparison of the efficacy of zolmitriptan and sumatriptan: issues in migraine trial design.* Cephalalgia, 2000. **20**(1): p. 30-8.

96. Goadsby, P.J., *The 'Act when Mild' (AwM) study: a step forward in our understanding of early treatment in acute migraine.* Cephalalgia, 2008. **28 Suppl 2**: p. 36-41.

97. Goadsby, P.J., et al., *Oral sumatriptan in acute migraine.* Lancet, 1991. **338**(8770): p. 782-3.

98. Goldstein, J., C. Keywood, and G. Study, *Frovatriptan for the acute treatment of migraine: a dose-finding study.* Headache, 2002. **42**(1): p. 41-8.

99. Krymchantowski, A.V. and M.E. Bigal, *Rizatriptan versus rizatriptan plus rofecoxib versus rizatriptan plus tolfenamic acid in the acute treatment of migraine.* BMC Neurol, 2004. **4**: p. 10.

100. Krymchantowski, A.V., P.F. Filho, and M.E. Bigal, *Rizatriptan vs. rizatriptan plus trimebutine for the acute treatment of migraine: a double-blind, randomized, cross-over, placebo-controlled study.* Cephalalgia, 2006. **26**(7): p. 871-4.

101. Mathew, N.T., et al., *Comparative efficacy of eletriptan 40 mg versus sumatriptan 100 mg.* Headache, 2003. **43**(3): p. 214-22.

102. **The Oral Sumatriptan International Multiple-Dose Study Group**, *Evaluation of a multiple-dose regimen of oral sumatriptan for the acute treatment of migraine. The Oral Sumatriptan International Multiple-Dose Study Group.* Eur Neurol, 1991. **31**(5): p. 306-13.

103. Santanello, N.C., et al., *Improvement in migraine-specific quality of life in a clinical trial of rizatriptan.* Cephalalgia, 1997. **17**(8): p. 867-72; discussion 800.

104. Scott, R.J., et al., *Oral sumatriptan in the acute treatment of migraine and migraine recurrence in general practice.* Qjm, 1996. **89**(8): p. 613-22.

105. Göbel, H. and A. Heinze, *The Migraine Intervention Score - a tool to improve efficacy of triptans in acute migraine therapy: the ALADIN study.* Int J Clin Pract, 2011. **65**(8): p. 879-86.

106. Spierings, E.L. and C. Keywood, *Rapid responders to frovatriptan in acute migraine treatment: results from a long-term, open-label study.* Pain Med, 2009. **10**(4): p. 633-8.

107. Kelman, L., et al., *Treatment response and tolerability of frovatriptan in patients reporting short- or long-duration migraines at baseline.* Curr Med Res Opin, 2010. **26**(9): p. 2097-104.

108. Maassen VanDenBrink, A., et al., *Chromosomal localization of the 5-HT1F receptor gene: no evidence for involvement in response to sumatriptan in migraine patients.* Am J Med Genet, 1998. **77**(5): p. 415-20.

109. MaassenVanDenBrink, A., et al., *5-HT1B receptor polymorphism and clinical response to sumatriptan.* Headache, 1998. **38**(4): p. 288-91.

110. Malik, S.N., et al., *Acute migraine treatment: patterns of use and satisfaction in a clinical population.* Headache, 2006. **46**(5): p. 773-80.

111. Mathew, N.T., *Early intervention with almotriptan improves sustained pain-free response in acute migraine.* Headache, 2003. **43**(10): p. 1075-9.

112. Mehrotra, S., et al., *The phe-124-Cys and A-161T variants of the human 5-HT1B receptor gene are not major determinants of the clinical response to sumatriptan.* Headache, 2007. **47**(5): p. 711-6.

113. Schoenen, J., et al., *Self-treatment of acute migraine with subcutaneous sumatriptan using an auto-injector device: comparison with customary treatment in an open, longitudinal study.* Cephalalgia, 1994. **14**(1): p. 55-63.

114. Schoenen, J., et al., *Patient preference for eletriptan 80 mg versus subcutaneous sumatriptan 6 mg: results of a crossover study in patients who have recently used subcutaneous sumatriptan.* Eur J Neurol, 2005. **12**(2): p. 108-17.

115. Ahonen, K., et al., *A randomized trial of rizatriptan in migraine attacks in children.* Neurology, 2006. **67**(7): p. 1135-40.

116. Banerjee, M. and L.J. Findley, *Sumatriptan in the treatment of acute migraine with aura.* Cephalalgia, 1992. **12**(1): p. 39-44.

117. Barbanti, P., et al., *Rizatriptan in migraineurs with unilateral cranial autonomic symptoms: a double-blind trial.* J Headache Pain, 2012. **13**(5): p. 407-14.

118. Cady, R., et al., *Randomized, placebo-controlled comparison of early use of frovatriptan in a migraine attack versus dosing after the headache has become moderate or severe.* Curr Med Res Opin, 2004. **20**(9): p. 1465-72.

119. Cady, R.K., et al., *Allodynia-associated symptoms, pain intensity and time to treatment: predicting treatment response in acute migraine intervention.* Headache, 2009. **49**(3): p. 350-63.

120. Cady, R.K., et al., *Rizatriptan 10-mg ODT for early treatment of migraine and impact of migraine education on treatment response.* Headache, 2009. **49**(5): p. 687-96.

121. Dahlöf, C., C. Edwards, and A. Toth, *Sumatriptan injection is superior to placebo in the acute treatment of migraine--with regard to both efficacy and general well-being.* Cephalalgia, 1992. **12**(4): p. 214-20.

122. Ensink, F.B., *Subcutaneous sumatriptan in the acute treatment of migraine. Sumatriptan International Study Group.* J Neurol, 1991. **238 Suppl 1**: p. S66-9.

123. Freitag, F., et al., *Elimination of migraine-associated nausea in patients treated with rizatriptan orally disintegrating tablet (ODT): a randomized, double-blind, placebo-controlled study.* Headache, 2008. **48**(3): p. 368-77.

124. Freitag, F.G., et al., *Effect of pain intensity and time to administration on responsiveness to almotriptan: results from AXERT 12.5 mg Time Versus Intensity Migraine Study (AIMS).* Headache, 2007. **47**(4): p. 519-30.

125. Goldstein, J., et al., *Acetaminophen, aspirin, and caffeine versus sumatriptan succinate in the early treatment of migraine: results from the ASSET trial.* Headache, 2005. **45**(8): p. 973-82.

126. Jensen, K., et al., *Introduction of a novel self-injector for sumatriptan. A controlled clinical trial in general practice.* Cephalalgia, 1995. **15**(5): p. 423-9.

127. Russell, M.B., et al., *A randomized double-blind placebo-controlled crossover study of subcutaneous sumatriptan in general practice.* Cephalalgia, 1994. **14**(4): p. 291-6.

128. Winner, P., *Sumatriptan formulated with RT technology™ as early intervention for migraine in adolescents.* Journal of Pediatric Neurology, 2011. **9**(2): p. 169-175.

129. al Deeb, S., et al., *Sumatriptan treatment of acute migraine attacks in a Saudi population.* Clin Neurol Neurosurg, 1997. **99**(1): p. 23-5.

130. Jenzer, G., et al., *An open multi-centre Swiss study on efficacy, safety and tolerability of oral sumatriptan in the treatment of migraine under practice conditions.* Schweizer Archiv fur Neurologie und Psychiatrie, 2000. **151**(2): p. 69-73.

131. Schulman, E.A. and K.F. Dermott, *Sumatriptan plus metoclopramide in triptan-nonresponsive migraineurs.* Headache, 2003. **43**(7): p. 729-33.

132. Cady, R.K., et al., *Postmarketing migraine survey of frovatriptan: effectiveness and tolerability vs previous triptans, NSAIDs or a combination.* Curr Med Res Opin, 2009. **25**(11): p. 2711-21.

133. Charlesworth, B.R., et al., *Speed of onset and efficacy of zolmitriptan nasal spray in the acute treatment of migraine: a randomised, double-blind, placebo-controlled, dose-ranging study versus zolmitriptan tablet.* CNS Drugs, 2003. **17**(9): p. 653-67.

134. Gruffydd-Jones, K., C.A. Hood, and D.B. Price, *A within-patient comparison of subcutaneous and oral sumatriptan in the acute treatment of migraine in general practice.* Cephalalgia, 1997. **17**(1): p. 31-6.

135. McGinley, J.S., et al., *Evaluating Mean Level and Within-Person Consistency in Migraine Pain Intensity and Migraine-Related Disability for AVP-825 vs Oral Sumatriptan: Results from the COMPASS Study, A Randomized Trial.* Headache, 2019. **59**(7): p. 1002-1013.

136. Silberstein, S., et al., *Early Onset of Efficacy and Consistency of Response Across Multiple Migraine Attacks From the Randomized COMPASS Study: AVP-825 Breath Powered(®) Exhalation Delivery System (Sumatriptan Nasal Powder) vs Oral Sumatriptan.* Headache, 2017. **57**(6): p. 862-876.

137. Jamieson, D., et al., *Real-world experiences in migraine therapy with rizatriptan.* Headache, 2003. **43**(3): p. 223-30.

138. Solomon, S., et al., *Migraine treatment outcomes with rizatriptan in triptan-naive patients: a naturalistic study.* Clin Ther, 2001. **23**(6): p. 886-900.

139. Visser, W.H., et al., *Sumatriptan-nonresponders: a survey in 366 migraine patients.* Headache, 1996. **36**(8): p. 471-5.

140. *Treatment of migraine attacks with sumatriptan.* N Engl J Med, 1991. **325**(5): p. 316-21.

141. *Self-treatment of acute migraine with subcutaneous sumatriptan using an auto-injector device. The Sumatriptan Auto-Injector Study Group.* Eur Neurol, 1991. **31**(5): p. 323-31.

142. Cady, R.K., et al., *Treatment of mild headache in disabled migraine sufferers: results of the Spectrum Study.* Headache, 2000. **40**(10): p. 792-7.

143. Cady, R.K., et al., *Effect of early intervention with sumatriptan on migraine pain: retrospective analyses of data from three clinical trials.* Clin Ther, 2000. **22**(9): p. 1035-48.

144. Freitag, F.G., et al., *Effect of pain intensity and time to administration on responsiveness to almotriptan: results from AXERT 12.5 mg Time Versus Intensity Migraine Study (AIMS).* Headache, 2007. **47**(4): p. 519-30.

145. Goadsby, P.J., et al., *Early vs. non-early intervention in acute migraine-'Act when Mild (AwM)'. A double-blind, placebo-controlled trial of almotriptan.* Cephalalgia, 2008. **28**(4): p. 383-91.

146. Visser, W.H., et al., *Treatment of migraine attacks with subcutaneous sumatriptan: first placebo-controlled study. The Subcutaneous Sumatriptan International Study Group.* Cephalalgia, 1992. **12**(5): p. 308-13.

147. Hu, X.H., et al., *Treatment of migraine with rizatriptan: when to take the medication.* Headache, 2002. **42**(1): p. 16-20.

148. Landy, S.H., et al., *A cross-sectional survey to assess the migraineur's medication decision-making beliefs: determining when a migraine is triptan-worthy.* Headache, 2013. **53**(7): p. 1134-46.

149. Leone, M., C. Vila, and C. McGown, *Influence of trigger factors on the efficacy of almotriptan as early intervention for the treatment of acute migraine in a primary care setting: the START study.* Expert Rev Neurother, 2010. **10**(9): p. 1399-408.

150. Linde, M., A. Mellberg, and C. Dahlöf, *Subcutaneous sumatriptan provides symptomatic relief at any pain intensity or time during the migraine attack.* Cephalalgia, 2006. **26**(2): p. 113-21.
